# High-throughput plant phenotyping identifies and discriminates biotic and abiotic stresses in tomato

**DOI:** 10.1016/j.plaphe.2025.100124

**Published:** 2025-09-30

**Authors:** Maria Isabella Prigigallo, Giovanni Bubici, Giorgia Batelli, Antonello Costa, Monica De Palma, Maria Teresa Melillo, Angelo Petrozza, Alessandra Ruggiero, Giorgia Sportelli, Stephan Summerer, Pasqua Veronico, Francesco Cellini, Marina Tucci, Livia Stavolone, Stefania Grillo, Fabrizio Cillo

**Affiliations:** a Istituto per la Protezione Sostenibile delle Piante, Consiglio Nazionale delle Ricerche, via Amendola 165/A, 70126, Bari, Italy; b Institute of Biosciences and Bioresources, National Research Council of Italy, via Università, 133, Portici, 80055, Napoli, Italy; c Agenzia Lucana Sviluppo & Innovazione in Agricoltura (ALSIA), Centro di Ricerche Metapontum Agrobios, S.S. 106 Jonica, km 448.2, Bernalda, 75010, Matera, Italy

**Keywords:** High-throughput plant phenotyping (HTP), *Solanum lycopersicum*, Precision agriculture, Remote sensing, Tomato spotted wilt virus (TSWV), Corky root rot, *Pseudopyrenochaeta lycopersici*, Root-knot nematode, *Meloidogyne incognita*, Drought

## Abstract

In the context of precision agriculture, high-throughput phenotyping (HTP) aims to rapidly and effectively identify factors that affect crop yield, enabling timely and appropriate interventions. However, interpreting data from HTP remains challenging. We performed a proximal red-green-blue (RGB)-based HTP on several tomato genotypes exposed to abiotic stress (drought) or biotic stress induced by tomato spotted wilt virus (TSWV), *Pseudopyrenochaeta lycopersici* (corky root rot; CRR), or *Meloidogyne* incognita (root-knot nematode; RKN). We aimed to determine if RGB-based HTP is effectively able to: a) distinguish the effects of biotic from abiotic stress; b) differentiate resistant/tolerant from susceptible genotypes. Our HTP data analysis produced 12 morphometric and eight colorimetric indices. Principal Component Analysis (PCA; *P* ​< ​0.0001; 83 ​% variation explained by three PCs) showed that factors such as shoot area solidity and certain color-based indices, including the senescence index and green area, effectively differentiated biotic from abiotic stress. Morphometric parameters, including plant height, projected shoot area, and convex hull area, proved to be applicable for identifying the stress status regardless of the type of stress. HTP effectively distinguished the genotype resistant to TSWV from the susceptible ones. This task was more challenging for below-ground stresses like CRR and RKN. Different profiles of HTP indices were observed among the genotypes assayed for drought tolerance, indicating variability in their ability to withstand drought conditions. In conclusion, our findings highlight the value of RGB-based HTP as a tool for precision farming of tomatoes, enabling the identification of both biotic and abiotic stressors.

## 1 Introduction

Modern agriculture is focused on enhancing both its economic and environmental sustainability. The European Union Green Deal aims to reduce the use of chemical pesticides, antimicrobials for farmed animals, and nutrient losses by 50 ​% by the year 2030 [1]. Precision farming greatly contributes to optimizing agricultural practices and reducing external inputs, with high-throughput phenotyping (HTP) being an important tool in this process. Over the last decades, HTP has primarily been developed and adopted in the United States of America, the European continent, and China. At the same time, HTP is valuable in modern plant breeding techniques [2]. Sometimes coupled with machine learning, it has been extensively applied in rice, wheat, and maize breeding programs to develop varieties that can tolerate abiotic stresses such as drought and salinity [3,4]. Numerous field, aerial, and unmanned platforms for HTP have recently been developed worldwide [5,6], while international facilities and networks have emerged for coordinated actions [7]. Machine learning and artificial intelligence are increasingly integrated into phenotyping workflows to manage and analyze the big data generated from HTP [8,9].

Research works dealing with HTP have grown rapidly in recent years, with a broader range of crops being explored. In tomato, canopy architecture can be adequately assessed using current HTP technology, e.g., 2D and 3D scanning techniques to reconstruct and measure tomato canopies [10], and deep-learning models to identify nodes, fruits, and flowers [11]. For this crop, Unmanned Ground Vehicles (UGVs) collecting RGB-D and multispectral images [12] and Unmanned Aerial Systems (UASs) [13] have contributed to monitoring key architectural traits. Adsorbed irradiance derived from thermal camera data [14] and a prototype of an electric phenomobile equipped with a multispectral camera have enabled the collection of many morphological and physiological traits in the field [15]. Even X-ray computed tomography has been applied in tomatoes to model 4D structural root architecture [16].

Several studies have demonstrated that drought stress can be identified in tomatoes using different methods and platforms. Unmanned Aerial Vehicles (UAVs) coupled with multispectral sensors are a reliable technology for phenotyping large tomato collections under water-deficit conditions in the field [17]. Both RGB and hyperspectral imaging have also been used to effectively describe the drought stress of pot-grown tomatoes in experimental facilities or platforms such as PlantScreen™ [18] and Scanalyzer 3D (LemnaTec) [[19], [20], [21]]. A *Solanum pimpinellifolium* germplasm collection has been screened for salinity tolerance using a LemnaTec platform as well as in the field using a UAV equipped with hyperspectral, thermal, and RGB cameras [22]. In contrast, HTP of tomato plants under biotic stress has been poorly investigated, with only a few field studies available. Notable examples include research focused on late blight (*Phytophthora infestans*), which utilized ground robots coupled with deep-learning algorithms [23] and UAVs equipped with multispectral cameras [24]. The combined use of RGB and multispectral cameras has yielded a strong correlation between visual estimation of disease symptoms and image-based indices in tomato and other vegetables under RKN attacks [25]. Patrick et al. [26] identified a set of multispectral indices that correlated well with the visual disease ratings on TSWV-infected peanut plants, demonstrating that image-based phenotyping can effectively identify genotype resistance.

In this work, high-throughput phenotyping was employed to recognize and differentiate between biotic and abiotic stresses in tomatoes, an aspect that has been poorly investigated so far. Specifically, we challenged several pot-grown tomato genotypes separately with five stressors and performed RGB-based HTP in a Scanalyzer 3D system. We report how RGB-based HTP effectively provides quantitative information on stress effects and how a selection of image-derived indices can predict, to some degree of specificity, the causal relationship between the stress and the derived phenotype modifications.

## 2 Materials and methods

Five experiments were conducted, each focusing on a different stress: tomato spotted wilt virus (TSWV; *Orthotospovirus tomatomaculae*), corky root rot (CRR), caused by *Pseudopyrenochaeta lycopersici*, the root-knot nematode (RKN) *Meloidogyne incognita*, and drought in two different seasons, spring and fall. Each experiment involved several plant genotypes along with a common variety, UC82 (Table S1). In specific experiments, such as those involving TSWV and CRR, the tomato genotypes were selected based on their known susceptibility and served as susceptible or resistant controls. The characteristics of all the genotypes are detailed in Table S2. Plants were transplanted into 2-liter polypropylene pots and grown in a glasshouse under natural photoperiod conditions at the ALSIA Metapontum Agrobios Research Centre, located in Metaponto, Matera, Italy (N 40° 23’ E 16° 47’). The experiments were conducted in separate greenhouses. The timing of the experiments, as well as the environmental temperature and relative humidity, are presented in Fig. S1.

### 2.1 Tomato spotted wilt virus (TSWV)

Four different tomato genotypes were selected for the experiment (Table S1): UC82 and the commercial hybrids Faber F_1_, which are susceptible to all TSWV isolates; Impact F_1_, carrying the *Sw-5* gene for resistance to TSWV but susceptible to mutant *Sw-5* resistance-breaking (SRB) isolates; and Dobler F_1_, also carrying the *Sw-5* gene and additionally displaying an intermediate resistance to TSWV SRB isolates. Twelve tomato plants per genotype were inoculated with the SRB TSWV isolate T-1012 [27].

The second and third true leaves of four-week-old plants were mechanically rubbed with virus-infected leaf tissues from young tomato plants, ground in a 1:20 (w/v) ratio in 0.1 ​M phosphate buffer pH 7.2 containing 0.2 ​% (w/v) Na_2_SO_3_ and 0.01 ​% β-mercaptoethanol. Plants inoculated with buffer only (mock-inoculated) were used as the negative controls.

Viral symptoms were visually evaluated at 6, 10, 16, and 20 days post-inoculation (dpi), and disease severity was assessed according to an empirical scale ranging from class 0 (no symptoms) to class 4 (severe necrosis on systemically infected leaves and stems). The disease severity was determined using the McKinney index (%), calculated as follows: [sum (class frequency ​× ​score of rating class)]/[(total number of plants) ​× ​(maximal disease index)] ​× ​100.

To monitor virus infection, leaves from nine inoculated plants of cv. UC82 were sampled at 10 and 16 dpi. They were pooled into three biological replicates, each consisting of three plants. Total RNA extraction, cDNA preparation, and RT-qPCR were made according to Bubici et al. (2017), using the primer pair TSWV-CP-17F and TSWV-CP-100R [28]. Data from the HTP platform were collected at 6, 10, 16, and 20 dpi.

### 2.2 Corky root rot (CRR)

The experiment involved two tomato genotypes: UC82 (susceptible) and Moboglan (resistant; Table S1). The soil used for the experiment was artificially inoculated with the *Pseudopyrenochaeta lycopersici* strain IPSP-GB485 following the method described by Bubici et al. [29] with slight modification. The fungus was cultured on potato dextrose agar (PDA) plates and incubated at 19 ​°C for 30 days. Then, the colonies were fragmented using a laboratory Waring blender and suspended in sterile distilled water. The amount of Colony Forming Units (CFUs) was determined by the dilution plate technique on PDA. Thirty-day-old tomato seedlings were transplanted into pots filled with heat-sterilized soil, either inoculated with 5×10^3^ ​*P. lycopersici* CFUs ∙ cm^−3^ of soil or left as a control. The heat-sterilization of 20 ​kg batches of soil was conducted in an oven at 80 ​°C for 24 ​h, one month before use. A randomized complete block design was employed, consisting of three blocks and five replicates (plants) per block, resulting in a total of 60 plants (2 genotypes ​× ​2 treatments ​× ​3 blocks ​× ​5 replicates). HTP was performed at 19, 28, and 50 dpi. Additionally, 15 extra plants were used to monitor the disease progression at 60, 90, and 120 dpi, with five plants assessed at each time point. The disease severity in the 60 plants was evaluated at 120 dpi. The severity of the corky root was assessed by visually estimating the percentage of roots affected by the symptoms, following the methodology outlined by Bubici et al. [29]. For this assessment, plants were uprooted, and the root systems were thoroughly washed with tap water to remove soil.

### 2.3 Root-knot nematodes (RKN)

The experiment was conducted with three tomato genotypes susceptible to RKN: UC82, San Marzano nano, and Regina di Fasano (Table S1). Plants were divided into two groups, each with twelve biological replicates (one per pot): i) no infestation, ii) RKN infestation. Seeds were germinated in quartz sand for six days in the dark at 25 ​°C in a growth chamber. Seedlings were transferred to 50 ​mL pots containing sterile sandy soil and grown in a growth chamber at 25 ​°C (photoperiod: 16 ​h light/8 ​h dark) for approximately three weeks, with daily watering using the Hoagland’s solution. Twenty-two-day-old plants were finally transplanted into 2 ​L polypropylene pots using the same soil mixture and grown in a glasshouse for an additional 15 days, until their root systems had developed well. Finally, 40-day-old plants were inoculated with 6000 ​*M. incognita* freshly hatched juveniles [30] by adding aliquots of the aqueous nematode suspension into two holes at the base of each plant. Non-inoculated plants served as controls. At 20, 40, and 60 dpi, three inoculated plants for each plant group and time point were uprooted to count the number of galls. Moreover, at the end of the experiment (60 dpi), the number of eggs, as well as the fresh weights of shoots and roots, were determined. For counting the number of eggs, roots were cut into small pieces and homogenized in a blender containing 1 ​% aqueous NaOCl. The water suspension was further sieved according to Thligene et al. [31]. Eggs collected from the last sieve were counted under a stereomicroscope Nikon SMZ745 (Nikon, Japan). Image capturing for HTP was done at 15, 19, 22, 26, 29, 33, and 62 dpi.

### 2.4 Drought

The drought experiment (spring) was conducted from May to June 2021 on 15 tomato genotypes of commercial importance at the national or regional level (Table S1). One week after transplanting, plants were divided into two groups: control and stressed plants. Drought was imposed by two consecutive cycles of water stress (DSI and DSII), each followed by a recovery phase, as previously described [32,33]. Drought stress consisted of daily irrigation at 30 ​% of the field capacity, as determined in control pots. DSI and DSII lasted 15 and 8 days, respectively. They were followed by two recovery stages (RWI and RWII), lasting 7 and 9 days, respectively, with daily irrigation at 100 ​% field capacity. Control plants were constantly irrigated at 100 ​% field capacity. A completely randomized block design was used with 6 replicates (plants) per genotype and treatment. HTP was carried out twice a week from eight to 41 days post-transplanting (dpt). Leaf proline content, a well-established indicator of water deficit, was determined at the end of DSI and RWI using the method described by Claussen [34]. At the end of RWII, fresh and dry shoot biomass accumulation, fruit number, and weight were determined.

Another drought experiment was established in the fall of 2021 (September and October), with two drought and rewatering cycles as described above. In this experiment, the genotypes UC82, M82, San Marzano nano, and Red Setter were used (Table S1). DSII was imposed with irrigation at 20 ​% field capacity for six days. HTP was carried out twice a week from 17 to 55 dpt. At the end of RWII, the shoot dry weight was determined.

### 2.5 High-throughput plant phenotyping (HTP) and image data analysis

Scanalyzer 3D platform (LemnaTec GmbH, Aachen, Germany) was used for the high-throughput plant phenotyping [35,36]. Only the visible light camera (Basler Scout scA1600-14gc) was used to take three orthogonal 1234 ​× ​1624 pixel images per plant, i.e., from the top, from the side with 0° and 90° rotation. Image analysis was performed using Python (v3.10) and PlantCV version 3.13 [37]. Pixels pertaining to plants were extracted or segmented via the following PlantCV functions: ‘plantcv.threshold.custom_range’, ‘plantcv.opening’, ‘plantcv.fill_holes’, and ‘plantcv.fill’. Contours of identified pixel blobs (plantcv.find_objects) were filtered (pcv.roi_objects using the ‘partial’ option) according to a region of interest (ROI) at the expected locations of plants (plantcv.roi.rectangle). Finally, the resulting object contours were united into a single object (pcv.object_composition), ready for analysis. Plant contour objects were analyzed with the following PlantCV functions: ‘plantcv.analyze_shape’ for the shoot area and solidity, ‘plantcv.analyze_bound_horizontal’ for plant height, and ‘plantcv.analyze_color’ for hue histogram data and hue circular mean. Data obtained with PlantCV were elaborated with R (version 4.3). Our pipeline yielded 20 HTP indices, including 12 morphometric and eight colorimetric. It was developed and improved over time in our facility [21,36,38]. Morphometric indices were plant height, plant height above reference, shoot width, shoot area, shoot convex hull area (area of the polygon that contains the canopy), and shoot area solidity (compactness of the canopy) [36]. Colorimetric indices were green area, greener area, hue circular mean, and senescence index. All these indices were recorded for the top and side of the plant canopy, except for plant height above reference, which was recorded only from the side view. Green area, greener area, and senescence indices were calculated from the hue channel histogram data as described by Zaman-Allah et al. [39]. Another index, the projected shoot area, was calculated as the sum of the shoot areas from the tree orthogonal images and subsequently converted from pixel^2^ to cm^2^ [21].

Finally, water use efficiency (WUE) was calculated as the irrigation water consumed per unit or projected shoot area increment during the entire experiment.

### 2.6 Statistical analysis

All the data were elaborated and statistically analyzed by using R 4.4.1 (ISBN 3-900051-07-0; http://www.Rproject.org) within RStudio 2024.09.0 build 375 (http://www.rstudio.com). Analysis of variance (ANOVA) was performed on the data separately by experiment, genotype, time point, and HTP index, after checking the assumptions for normality and heteroscedasticity. When the ANOVA *P* value was significant (<0.05), the means of control and stress conditions were compared using the Student’s *t-*test, and genotypes were compared using the Tukey test. Plots were generated by *ggplot2* package v. 3.5.1.

Principal Component Analysis (PCA) was performed by the *prcomp* function of the *stats* package, with plots generated by *ggbiplot* package v. 0.55. This analysis was conducted on data calculated as the stress-to-control condition ratio for each HTP index, stress, genotype, and time point. It made the data from the five experiments comparable. Data from early time points were excluded from this analysis to better visualize the differences between treated and control groups of plants, which became more significant as the experiments progressed. In particular, time points earlier than the following values were excluded: 10 dpi for TSWW, 22 dpi for RKN, 27 dpt for spring drought, and 30 dpt for fall drought experiments. PCA robustness was tested using the *PCAtest* package with 1000 bootstrap replicates and 1000 random permutations.

## 3 Results

### 3.1 Tomato responses to the stresses imposed

The development of TSWV T-1012 symptoms was visually estimated in four different tomato genotypes over 24 days, a period also covered by HTP. While no symptoms were visible in the earliest phase of infection, the first symptoms appeared at 10 dpi in all four genotypes, with light leaf bronzing and anthocyanin accumulation visible on both leaf sides. From 16 dpi, plants displayed more genotype-specific evident symptoms. ‘UC82’ plants were more prone to leaf bronzing (Fig. S2A and S2E), whereas ‘Faber F_1_’ plants, also lacking the *Sw-5* resistance gene to TSWV, displayed leaf yellowing with brown necrotic spots, later merging in wider leaf areas (Fig. S2B). ‘Impact F_1_’ plants, carrying the *Sw-5* resistance gene but susceptible to the TSWV SRB isolate T-1012, displayed more severe symptoms, including extended necrotic areas on leaves, petioles, and stems (Fig. S2C and S2E). Most ‘Dobler F_1_’ plants did not display visible symptoms, although four out of 12 plants developed symptoms of severe necrosis on systemically infected leaves and stems (Fig. S2D). The appearance and increase of viral symptoms over time correlated with the progress of infection, i.e., the virus’s systemic invasion and replication. TSWV RNA accumulation levels showed a more than 100-fold increase at 16 vs. 10 dpi in ‘UC82’ plants (Fig. S2G).

A high CRR disease pressure was observed in this study (Fig. S3). Root symptoms appeared on the susceptible genotype UC82 at 28 dpi (severity of approximately 5 ​%; Fig. S3A). Their severity rose to 70 ​% at 61 dpi and 75 ​% at 91 dpi. Under this high pathogen pressure, the resistant genotype Moboglan was not free from CRR; the symptom severity reached 25 ​% at 91 dpi in this genotype. Inoculated plants also showed leaf yellowing (Fig. S3B).

RKN infestation resulted in a progressive increase in the number of detectable galls at 20, 40, and 60 dpi, a hallmark of a successful infestation and development of a second nematode generation (Fig. S4).

In the spring drought experiment, plants subjected to drought stress showed drastic biomass reduction (e.g., ranging from 16 to 34 ​% for shoot fresh weight, and from 39 to 88 ​% for weight of fruits), which was statistically significant for many genotypes (Fig. S5A). Fruit number and/or weight were also reduced in drought-stressed plants in several genotypes, including Red Setter, UC82, Cerise, and Torremaggiore (Fig. S5A). Leaf proline content showed a significant (*P* ​< ​0.05) increase in ‘Red Setter’, ‘Torremaggiore’, and ‘Dobler F_1_’ in stressed compared to control plants at the end of the first drought stress cycle. By contrast, at the end of the first rewatering cycle, proline levels were similar in control and stressed plants, except for ‘Dobler F_1_’, indicating a recovery from the stress (Fig. S5B).

In the fall drought experiment, a substantial plant biomass reduction was observed at the end of the second rewatering stage (Fig. S6A and S6B). Shoot dry weight was significantly reduced in ‘UC82’, ‘Red Setter’, and ‘San Marzano nano’ in stressed compared to well-watered plants (Fig. S6A).

### 3.2 HTP allowed the identification of stressed plants

The entire dataset, including 20 HTP indices collected and elaborated from the stress experiments described above, is available in Table S3.

**Fig. 1:**
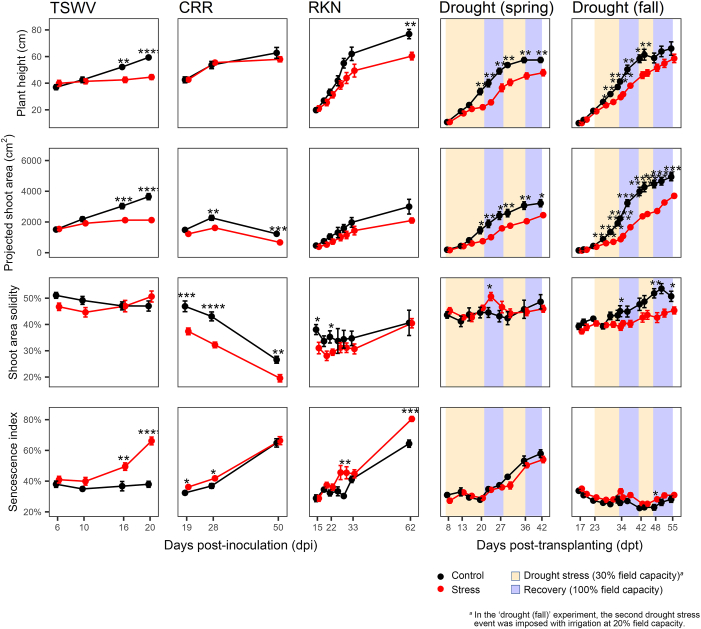
Progress over time of four HTP indices on tomato plants cv. UC82 challenged separately with five stresses: tomato spotted wilt virus (TSWV), corky root rot (CRR), root-knot nematode (RKN), and drought conducted in spring and fall. Error bars indicate the standard error of the mean. Asterisks indicate a significant difference between control and stressed plants according to the Student’s *t*-test (∗ ​= ​*P* ​< ​0.05; ∗∗ ​= ​*P* ​< ​0.01; ∗∗∗ ​= ​*P* ​< ​0.001; ∗∗∗∗ ​= ​*P* ​< ​0.0001).

First, we analyzed four indices that were informative of the whole phenotyping: plant height, projected shoot area, shoot area solidity, and senescence, all taken from the side view of the plants. Indeed, many indices were correlated with each other (Fig. S7) or included other indices in their calculation. Plant height, for instance, gives an easy idea of plant development and was highly correlated with the other morphometric indices. The projected shoot area incorporated the shoot areas from one top and two side views. Shoot area solidity had among the weakest correlations with the other indices. The senescence index was representative of the colorimetric indices because it was highly negatively correlated with them. Fig. 1 shows the progress of the four selected HTP indices in ‘UC82’ throughout the five experiments. Differences between stressed and control plants were more evident at later stages of the experiment, suggesting a cumulative negative effect over time of the stress on plant growth and physiology. Plant size, described by height and projected shoot area, was significantly reduced in plants challenged with TSWV and drought. The size reduction was less evident in plants subjected to root stresses, such as CRR and RKN, as only one of these two indices was significantly (*P* ​< ​0.05) affected. Shoot area solidity, indicating the compactness of the plant canopy, was not effective in discriminating stressed from control plants, as a significant difference was detected only at specific time points and for some stressors (e.g., for CRR at all time points, for RKN at 15 and 22 dpi, and fall drought experiment at 34, 48, 51, and 55 dpi). In these cases, stressed plants showed higher shoot area solidity values than the control. The senescence index was higher in stressed plants, although no significant difference was observed between treated and untreated plants during the spring drought experiment.

The progress of TSWV disease was fast, with early symptoms and severe foliar necrosis visible at 16 dpi. The curves of the HTP indices accurately described the plant phenotype, and significant differences were observed between stressed and control plants at 16 and 20 dpi for all indices except shoot area solidity, for which differences were not significant. Plants infected by TSWV showed stunted growth and early senescence, while the limited effect on shoot area solidity indicated that morphological parameters of the canopy, such as leaf orientation and leaf blade surface, were not significantly altered by the virus.

For the two biotic root stresses CRR and RKN, the ability of HTP to discriminate stressed versus control plants was less effective compared to TSWV. Differences in plant height and projected shoot area were not always statistically significant. Inoculated plants showed higher values of shoot solidity and senescence. For RKN, peaks of the senescence index occurred at 26, 29, and 62 dpi, probably in conjunction with completing two consecutive biological cycles of the nematodes.

The detrimental effect of drought on plant height and projected shoot area was similar in the spring and fall experiments. In stressed plants, these parameters increased at lower rates during the drought periods rather than the recovery events, indicating that during the recovery stages, the plant growth rate tended to be restored to control conditions. Differences in the shoot area solidity and senescence index were observed between the two experiments, possibly due to different seasonal changes of environmental conditions, including maximum, minimum temperatures, and photoperiod (Fig. S1). In the fall drought experiment, stressed plants showed a less compact canopy than control plants, especially at later stages. Such a difference was not evident in the spring experiment. In both experiments, no significant difference occurred for the senescence index between stressed and control plants in ‘UC82’ (Fig. 1). In the other genotypes (average data), such a significant difference occurred in the spring experiment (Fig. S8).

Therefore, different HTP indices were variably effective in detecting the stress. TSWV symptom severity, for instance, showed strong (*P* ​< ​0.0001) correlation with colorimetric HTP indices such as the senescence index (ρ ​> ​0.71) or green area (ρ ​< ​−0.59) and weak or no correlation with morphometric indices such as area or height (Fig. S9). For CRR, symptom severity significantly correlated (*P* ​< ​0.05) with all HTP indices except for height and width (Fig. S10). For RKN, the senescence index was correlated with the number of root galls; hence, the other colorimetric indices were negatively correlated. However, the morphometric indices were positively correlated with root gall number, suggesting that RKN did not significantly affect the plant development under our experimental conditions (Fig. S11). Proline content, determined at the end of the first drought event on plants subjected to drought in the spring, was negatively correlated (*P* ​< ​0.0001) with morphometric indices (except for the solidity index from the side view) and, among the colorimetric indices, positively correlated (*P* ​< ​0.0001) only with hue circular mean calculated from the top view (Fig. S12).

### 3.3 HTP allowed the discrimination of biotic and abiotic stresses

**Fig. 2:**
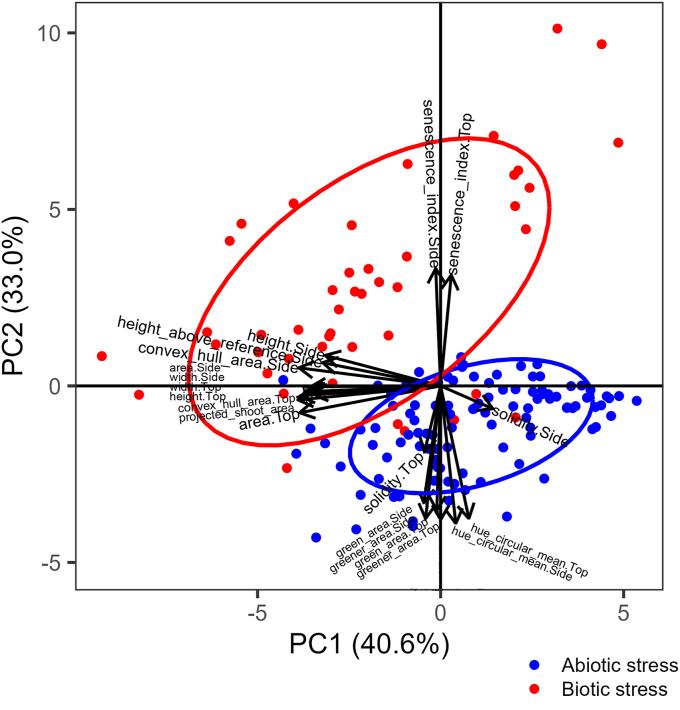
Principal Component Analysis (PCA) of data calculated as the ratio of stress to control condition per each HTP index, stress, genotype, and time point.

Principal Component Analysis (PCA), constructed with data calculated as the stress-to-control condition ratio for each HTP index, was highly significant (*P* ​< ​0.0001, empirical Ψ index ​= ​95.09, null hypothesis-Ψ index ranging from 1.98 to 3.689). Principal Components (PCs) 1, 2, and 3 were significant (Benjamini-Hochberg-adjusted *P* ​= ​0.0017) and accounted for 83.1 ​% of the total variation. Most of the morphometric indices had significant loadings on PC1 (e.g., height, area, width, projected shoot area), and most of the color-based indices on PC2 (e.g., green area, hue circular mean, senescence index). Senescence index (top) and solidity (side) also had significant loadings on PC3. PCA revealed a clear clustering of biotic and abiotic stresses, indicating that HTP could efficiently discriminate between these two types of stress (Fig. 2). Data points related to abiotic stresses were mainly located in the lower quadrants, along with low values of variables such as solidity, hue circular mean, and green area. Conversely, data points for biotic stresses were mainly located in the upper quadrants, characterized by high values of senescence indices and morphometric parameters, such as height, projected shoot area, and convex hull area. The senescence indices were in a perfect opposite direction to the hue circular mean, green area, and solidity, meaning that they formed two distinct index groups, particularly informative for discriminating biotic and abiotic stresses. Overall, this indicated that plants subjected to drought were greener and had a more compact canopy. Conversely, plants challenged with pathogens or nematodes experienced a more rapid senescence. Morphometric variables followed a direction almost orthogonal to the orientation of clusterization because challenged plants were smaller than the controls, independently of the applied stress. However, growth stunting was slightly more pronounced in biotic stresses.

**Fig. 3:**
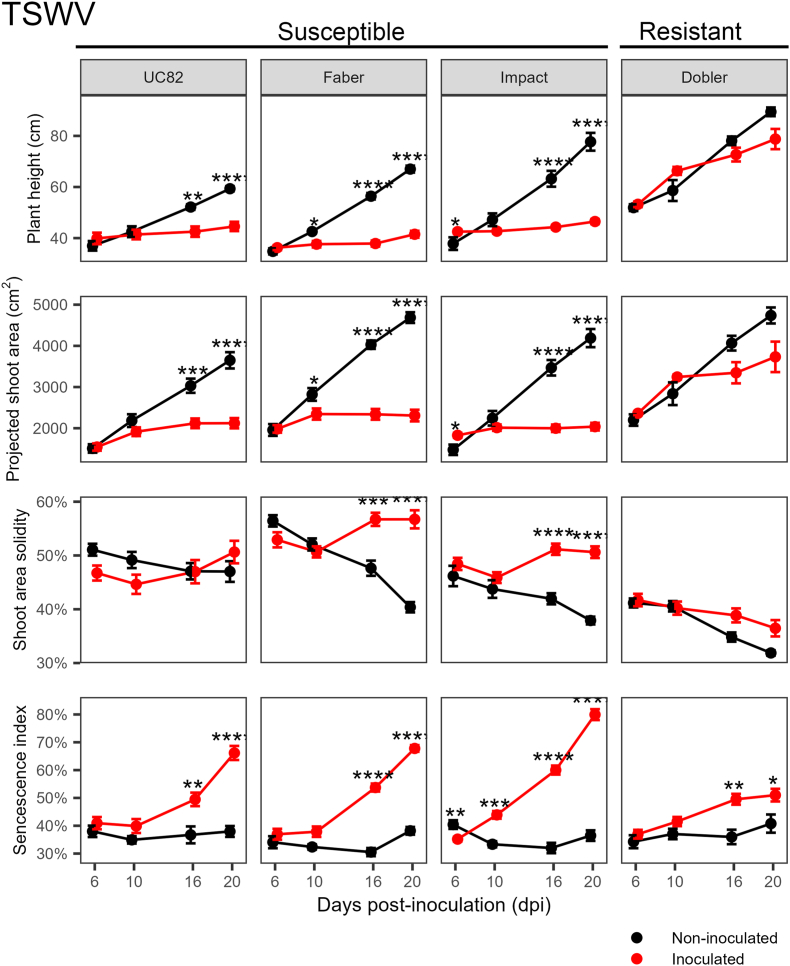
Progress over time of four HTP indices on four tomato genotypes with different susceptibility levels inoculated with tomato spotted wilt virus (TSWV). Error bars indicate the standard error of the mean (12 inoculated plants and 6 non-inoculated ones). Asterisks indicate a significant difference between control and stressed plants according to the Student’s *t*-test (∗ ​= ​*P* ​< ​0.05; ∗∗ ​= ​*P* ​< ​0.01; ∗∗∗ ​= ​*P* ​< ​0.001; ∗∗∗∗ ​= ​*P* ​< ​0.0001).

**Fig. 4:**
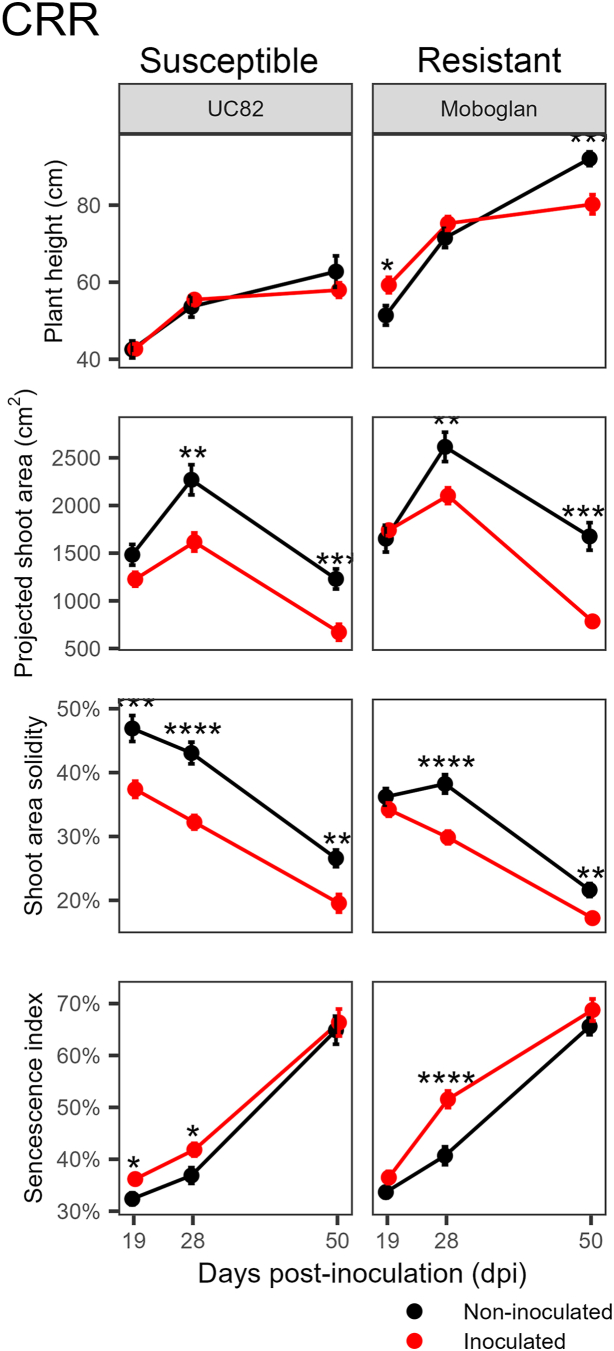
Progress over time of four HTP indices on two tomato genotypes with different susceptibility levels inoculated with *Pseudopyrenochaeta lycopersici*, the causal agent of corky root rot (CRR). Error bars indicate the standard error of the mean (n ​= ​15). Asterisks indicate a significant difference between control and stressed plants according to the Student’s *t*-test (∗ ​= ​*P* ​< ​0.05; ∗∗ ​= ​*P* ​< ​0.01; ∗∗∗ ​= ​*P* ​< ​0.001; ∗∗∗∗ ​= ​*P* ​< ​0.0001).

**Fig. 5:**
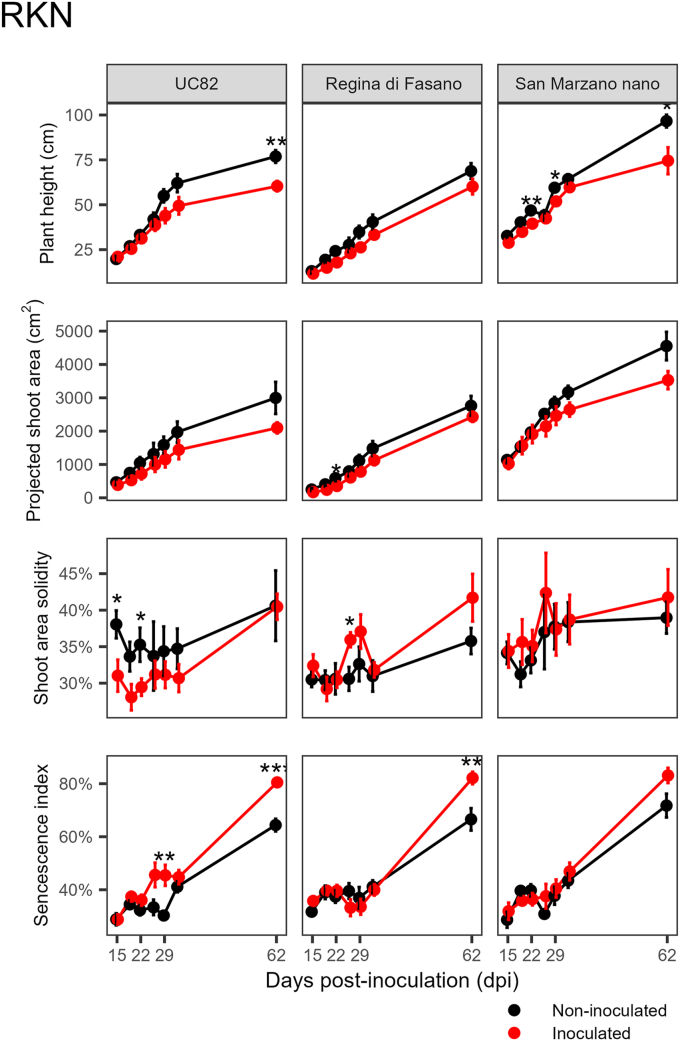
Progress over time of four HTP indices on three tomato genotypes with different susceptibility levels inoculated with the root-knot nematode (RKN) *Meloidogyne incognita*. Error bars indicate the standard error of the mean (n ​= ​12). Asterisks indicate a significant difference between control and stressed plants according to the Student’s *t*-test (∗ ​= ​*P* ​< ​0.05; ∗∗ ​= ​*P* ​< ​0.01; ∗∗∗ ​= ​*P* ​< ​0.001).

**Fig. 6:**
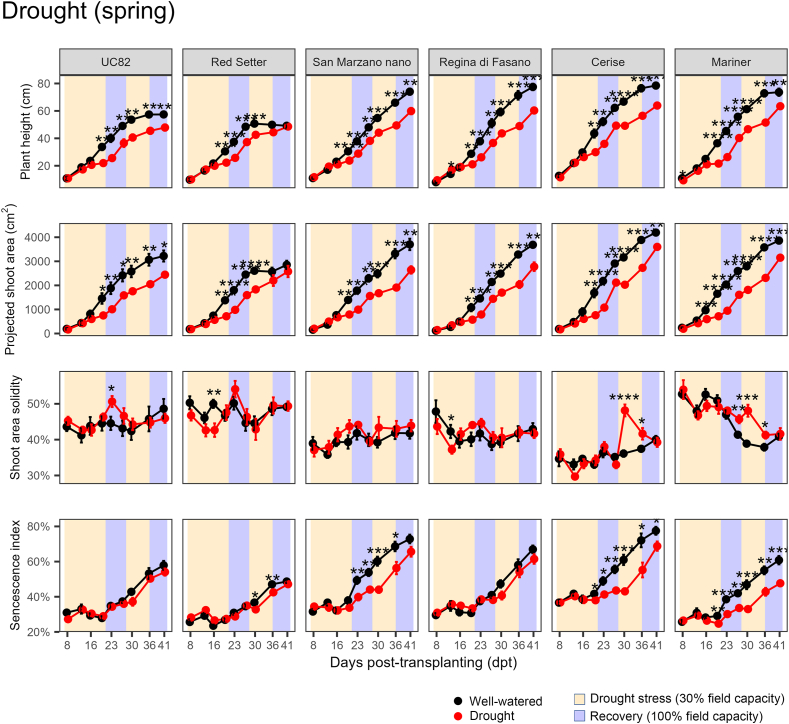
Progress over time of four HTP indices on six tomato genotypes subjected to two cycles of drought stress and recovery in the spring of 2021. Error bars indicate the standard error of the mean (n ​= ​6). Asterisks indicate a significant difference between control and stressed plants according to the Student’s *t*-test (∗ ​= ​*P* ​< ​0.05; ∗∗ ​= ​*P* ​< ​0.01; ∗∗∗ ​= ​*P* ​< ​0.001; ∗∗∗∗ ​= ​*P* ​< ​0.0001). Other genotypes are shown in Fig. S13.

**Fig. 7:**
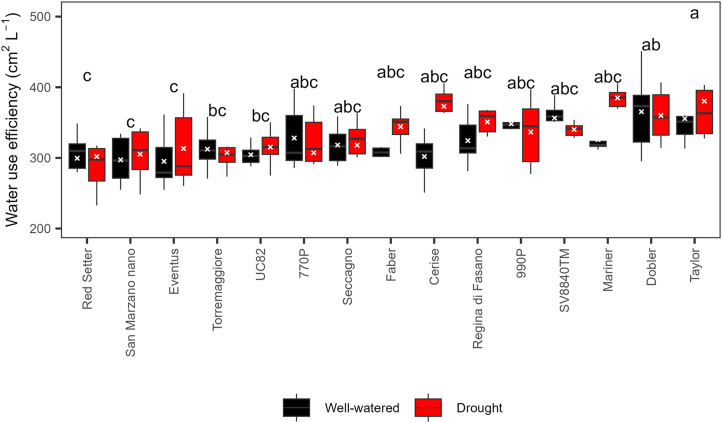
Water use efficiency of 15 tomato genotypes subjected to two cycles of drought stress and recovery in the spring of 2021. Genotypes with different letters are significantly different according to the Tukey test (*P* ​< ​0.05). The analysis of variance revealed significant effects for the genotype (*P* ​< ​0.0001) and treatment (*P* ​= ​0.383), but not their interaction (*P* ​= ​0.2074).

The plot in Fig. 2 also shows the correlations among the HTP indices, which correspond to the arrow directions. For example, all the morphometric parameters (e.g., heigh.Side, projected_shoot_ area, area.Top, etc.), except for solidity.Side, were in the same direction, orthogonal to that of the colorimetric indices (e.g., green_area.Side, hue_circular_mean.Side, etc.) and that of the senescence indices. Therefore, indices such as height, solidity, and senescence, which are in different directions, could be considered the most informative of the entire phenotyping, whereas the others provided redundant (correlated), though useful, information. With this consideration in mind, to present our data effectively and straightforwardly, we analyzed four of the 20 indices: plant height, projected shoot area, shoot area solidity, and senescence index (Fig. 1, Fig. 3, Fig. 4, Fig. 5, Fig. 6, Fig. 7). We report data from the other indices in the supplemental material (Table S3).

### 3.4 HTP allowed the discrimination of resistant and susceptible plant genotypes

The magnitude of the difference between stressed and control plants can indicate the susceptibility/resistance level of a given genotype: the larger this difference, the higher the susceptibility level.

In the TSWV experiment, at the last two time points, stressed and control plants of the resistant variety Dobler F_1_ did not differ significantly in height, projected shoot area, or solidity, while significant differences occurred in the susceptible genotypes UC82, Faber F_1_, and Impact F_1_ (Fig. 3). Differences in the senescence index were statistically significant for both susceptible and resistant genotypes, but were smaller in the latter.

For CRR, HTP indices were ineffective in discriminating susceptible and resistant genotypes (Fig. 4). For RKN, where only susceptible genotypes were investigated, the plant height index revealed that nematode infection led to a delay in plant growth (Fig. 5). The differences were statistically relevant for ‘UC82’ and ‘San Marzano nano’, which also showed a decrease in aerial part fresh weight in infected plants compared to uninfected ones (Fig. S4). The senescence index indicated the presence of stress from 29 dpi, after the first life cycle of the nematode. The subsequent root attack by the new generation of nematodes maintained high index values until the subsequent HTP capture at 62 dpi (Fig. 5).

In the two drought experiments, two groups of contrasting genotypes were identified. In the spring, for example, ‘UC82’ and ‘Red Setter’ showed smaller differences in the four HTP indices between stressed and control plants than ‘Cerise’ and ‘Mariner F_1_’ (Fig. 6). At 36 and 41 dpt, ‘Cerise’ stressed plants were significantly (*P* ​< ​0.01) smaller (i.e., lower plant height and projected shoot area) than the control, whereas ‘Red Setter’ stressed and control plants were statistically similar in size. Also, ‘Cerise’ stressed plants showed values of senescence index significantly higher than control plants from 19 to 41 dpt, whereas similar significant differences occurred only at 30 and 36 dpt in ‘Red Setter’ and at no time points in ‘UC82’ (Fig. 6). ‘San Marzano nano’ and ‘Regina di Fasano’ had intermediate responses between ‘UC82’ and ‘Mariner F_1_’ (Fig. 6). The ANOVA of WUE data revealed significant effects of genotype (*P* ​< ​0.0001) and drought treatment (*P* ​= ​0.383), but not their interaction (*P* ​= ​0.2074). ‘Taylor F_1_^’^, ‘Dobler F_1_^’^, and ‘Mariner F_1_^’^ were the genotypes with the highest WUE, in contrast with others such as ‘Red Setter’, ‘San Marzano nano’, and ‘UC82’ (Fig. 7). WUE did not correlate with proline content (*P* ​= ​0.20). As mentioned above, after the first drought event, proline significantly increased only in ‘Red Setter’, ‘Torremaggiore’, and ‘Dobler F_1_’. WUE correlated well with HTP indices, with positive R values for all except the senescence index (non-significant *P* values) and solidity (negative R values) (Fig. S14).

**Fig. 8:**
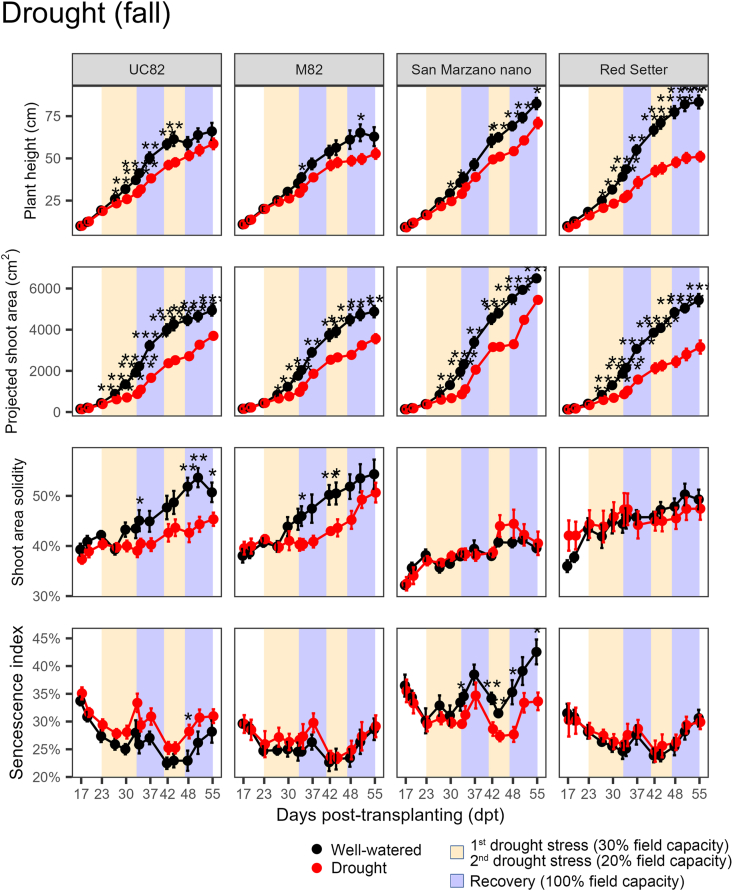
Progress over time of four HTP indices on four tomato genotypes subjected to two cycles of drought stress and recovery in the fall of 2021. Error bars indicate the standard error of the mean (n ​= ​6). Asterisks indicate a significant difference between control and stressed plants according to the Student’s *t*-test (∗ ​= ​*P* ​< ​0.05; ∗∗ ​= ​*P* ​< ​0.01; ∗∗∗ ​= ​*P* ​< ​0.001; ∗∗∗∗ ​= ​*P* ​< ​0.0001).

**Fig. 9:**
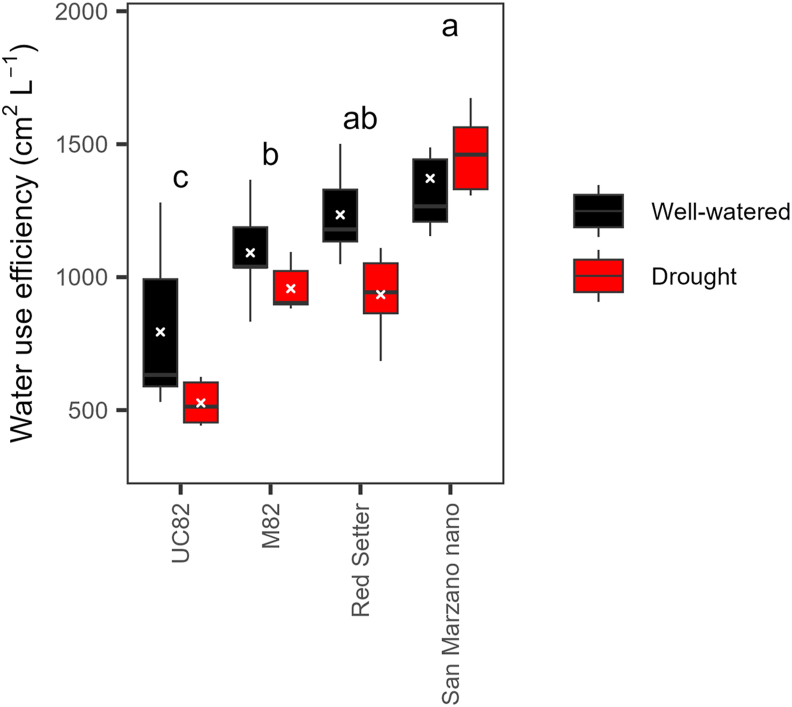
Water use efficiency of four tomato genotypes subjected to two cycles of drought stress and recovery in the fall of 2021. Genotypes with different letters are significantly different according to the Tukey test (*P* ​< ​0.05). The analysis of variance revealed significant effects for the genotype (*P* ​< ​0.0001) and treatment (*P* ​= ​0.0138), but not their interaction (*P* ​= ​0.7318).

In the fall, ‘UC82’ and ‘M82’ differed from ‘San Marzano nano’ and ‘Red Setter’ in plant height, projected shoot area, and shoot area solidity (Fig. 8). For example, at the end of the experiment, ‘Red Setter’ stressed plants were significantly (*P* ​< ​0.0001) shorter (plant height) than the control plants, whereas such a difference did not occur in ‘UC82’. However, all genotypes, except for ‘M82’, showed significant (*P* ​< ​0.01) reductions in shoot dry weight due to water deficit (Fig. S6). For the WUE, the effects of genotype and drought treatment were statistically significant (*P* ​< ​0.0001 and P ​= ​0.0138, respectively), with no significant interaction (*P* ​= ​0.7318). ‘UC82’ and ‘San Marzano nano’ were the most contrasting genotypes with WUE mean values of 660.28 and 1325.93 ​cm^2^ ​L^−1^, respectively (Fig. 9). Similar to the spring, in the fall, WUE showed significant and positive correlations with morphometric HTP indices. Among colorimetric indices, instead, the correlations were significant only for the greener area, hue circular mean (side), and senescence index (top) (Fig. S15). Plants grown in the spring accumulated less biomass than those grown in the fall, both under control and drought stress conditions (Figs. S5 and S6). This difference could arise from variations in temperature and relative humidity during the two growth cycles.

## 4 Discussion

Precision agriculture is increasingly recognized as a key strategy for improving the sustainability of modern agriculture. Through this research, conducted using a Scanalyzer 3D system, we aimed to determine whether RGB-based HTP could effectively identify stress status in tomato plants early, differentiate between types of stressors, and discriminate between resistant and susceptible plant genotypes. From RGB images, we analyzed 20 indices, four of which were identified as representative of the whole phenotyping because they were good indicators of the overall plant performance and described canopy size, shape, and color under the assayed conditions: plant height, projected shoot area, shoot area solidity, and senescence index. Monitoring these indices over the entire experiments allowed us to effectively identify plants stressed by a virus (TSWV), a fungus (*P. lycopersici*), a nematode (*M. incognita*), and drought in two seasons (spring and fall). Unsurprisingly, we observed a high effectiveness of HTP in detecting stresses such as TSWV and drought. In fact, TSWV causes growth stunting, wilting, yellowing, and necrosis, whereas drought induces growth stunting, wilting, and leaf greening, symptoms that are all detectable through an RGB camera. Several examples of phenotyping plants subjected to viral diseases exist, including one with TSWV in peanuts using RGB, multispectral, and thermal sensors mounted on a UAV [40] and other examples with diverse viruses and HTP technologies [[41], [42], [43], [44], [45], [46], [47], [48]]. In a study conducted on another viral disease, groundnut rosette virus (GRV), the authors utilized RGB images to collect data such as the green area, greener area, hue angle, and crop senescence index [47]. Unlike our approach, Chapu and colleagues (2022) did not calculate indices related to canopy size and shape; instead, they extracted the lightness, intensity, and saturation and calculated several indices based on the values of the red, green, and blue channels. This highlights the versatility of RGB images, which can be leveraged in various ways and at multiple levels to detect plant stress effectively. Drought tolerance has been extensively studied using HTP, both in tomatoes [[17], [18], [19],^21^,^49^,^50^] and several other crops [3,51,52], confirming the well-known feasibility of HTP for this purpose.

More interestingly, our above-ground HTP effectively recognized even stresses imposed on the below-ground plant part, including those caused by the soil-borne fungus *P. lycopersici* and the root nematode *M. incognita*, suggesting that RGB-based HTP of the tomato canopy can also reveal what happens below ground, where the human eye cannot see. Actually, we observed a lower effectiveness of HTP in detecting CRR and RKN compared to TSWV, as significant differences between stress and control plants occurred only on some indices and at certain time points. Additionally, discrimination between genotypes resistant or susceptible to CRR was not achieved, despite showing marked differences in disease severity. On the other hand, although yield losses of 20–50 ​% have been reported, CRR does not cause remarkable symptoms on the leaves, even when the roots are severely affected [53]. Canopy HTP has been proven effective in detecting pathogens and parasites affecting the roots, such as *Pythium ultimum* in lettuce, tomato, basil [54], and *Verticillium dahliae* in olives [55,56], though they are known to cause significant symptoms on the leaves. RKN is known to induce yellowing and stunting on tomatoes, symptoms that we detected by HTP only in one or two genotypes and at the last time point of the experiment, probably because the severity of the attack was not particularly high. We inoculated the soil with three juveniles per gram, following the same procedure as in other studies on tomato [30]. In *Arabidopsis thaliana*, the green canopy area has been significantly reduced when inoculated with at least 15 juveniles per gram of substrate [57].

We observed lower plant height and projected shoot area in stressed plants regardless of the type of stress (Fig. 1, Fig. 3, Fig. 4, Fig. 5, Fig. 6, Fig. 7), indicating that both biotic and abiotic stresses induced growth stunting. Many researchers have experienced that biometric measurements, regardless of the techniques or instruments employed, effectively illustrate changes in plant canopies resulting from various stresses or conditions [10,12,13,15,17]. Plants inoculated with TSWV, CRR, and RKN also encountered early senescence (higher senescence index values), whereas plants exposed to drought stress, especially in the spring experiment, exhibited leaf greening (lower senescence index values). In the fall experiment, leaf greening occurred only in one of the four assayed genotypes, suggesting that leaf greening induced by water stress was influenced by the season. A similar scenario of increased leaf greening (HUE index) of tomatoes under water stress has been observed in previous research conducted in the spring of 2021 [21]. Once again, another piece of evidence of leaf greening in tomatoes due to water stress has been derived from SPAD measurements, which correlate with the leaf chlorophyll content and, thus, the intensity of the green color [58]. An increase in the content of photosynthetic pigments has been reported in tomatoes, as well as in other species, under water deficit [59] and salt stress conditions [60].

The shoot area solidity index, which indicates the compactness of the canopy, was expected to describe well the stresses we considered. This is because the wilting of shoots caused by any stress should result in higher values of this index, indicating a denser canopy due to increased ratios of leaf-occupied to empty space pixels in the RGB images. This expectation was not fully met, except in some instances, such as when some genotypes were inoculated with TSWV (Fig. 3) or exposed to drought (Fig. 6). On the other hand, an opposite situation occurred for two genotypes challenged with CRR (Fig. 4) and two genotypes subjected to water stress in the fall experiment (Fig. 7). These plants showed a reduced shoot area solidity under stress compared to the control. Therefore, although theoretically reliable, this index did not perform well in our experiments. In rice, canopy compactness has been significantly lower in stressed than control plants, especially in a susceptible genotype compared to a drought-tolerant mutant [51]. In the same research, the susceptible genotype under drought stress showed a reduction in the green leaf area, which contrasts with our observation and suggests that the change in leaf color due to drought is likely dependent on the plant species.

Once we realized how our HTP method could help evaluate the stresses in tomato, we attempted to determine whether it could distinguish between different types of stressors. Principal component analysis (PCA) revealed the most intriguing finding of our research. Biotic and abiotic stresses clustered separately, indicating that RGB-based HTP could determine whether a plant was affected by biotic stress or drought (Fig. 2). Color-based indices such as the senescence index, green area, and hue circular mean were good indicators of the type of stress affecting the plants. Overall, the biotic stresses TSWV, CRR, and RKN caused early senescence of tomato leaves, whereas drought led to their greening. Similarly, shoot area solidity served as another indicator of the type of stress, as plants under drought had a denser canopy compared to those challenged by TSWV, CRR, or RKN. Size-related indices, such as plant height, projected shoot area, and convex hull area, effectively identified stressed plants, regardless of the type of stress, as all stress types caused growth stunting. To our knowledge, this is some of the first evidence that HTP is not only effective in detecting stress in plants but also in identifying the type of stress. In previous research, the dynamics of chlorophyll fluorescence have proven effective in classifying plants as healthy or unhealthy across several crops subjected to biotic stress, such as root infection by *Pythium ultimum* or the leaf pathogen *Podosphaera aphanis* [54]. Those results were less encouraging for abiotic stress, such as drought and salinity, and no attempts were made to identify the specific type of stress, as was done in our study. A machine learning algorithm using aerial hyperspectral and thermal imagery has effectively distinguished between symptoms caused by two vascular pathogens in olive trees, *Xylella fastidiosa* and *Verticillium dahliae* [56]. The most discriminating spectral traits were the blue index B, the leaf inclination distribution function (LIDF), and the carotenoid pigment content. Furthermore, airborne spectroscopy and thermal scanning have effectively differentiated *V. dahliae* and *X. fastidiosa* symptoms in olives and uncoupled biotic-abiotic spectral dynamics in irrigated and non-irrigated almond orchards affected by *X. fastidiosa* [55]. For detecting *X. fastidiosa* symptoms, for example, the normalized phaeophytinization index (NPQI) and anthocyanin content were very helpful in olive trees but of limited importance in almonds. Hyperspectral imaging has also proven effective in the early detection of root-knot nematode infestations and in distinguishing them from water deficiency in tomatoes [61,62].

We also demonstrated that differences in the susceptibility level can be appraised in tomato plants using RGB-based HTP. The TSWV experiment clearly showed that the susceptible varieties UC82, Faber F_1_, and Impact F_1_ could be differentiated from the resistant hybrid Dobler F_1_ based on any HTP index (Fig. 3). Nevertheless, this goal was not achieved for CRR and RKN, whose causal agents act on the root system (Fig. 4, Fig. 5). We identified some genotypes with previously unknown contrasting responses to drought. However, the results from HTP and the water use efficiency data were in opposite directions, thus preventing us from drawing definitive conclusions regarding the drought tolerance. We did not observe any evident suffering in tomato plants due to the pot size (2 ​L), which has been reported as a limiting factor when comparing drought tolerance in oilseed rape grown in pots of 220 ​mL versus 6 ​L [63]. Based on a hyperspectral index (H-index), projected shoot area, hue angle, and senescence index, tomato varieties Red Setter and Torremaggiore showed levels of drought tolerance higher than 770P and 990P [21]. These results align with ours when we consider reductions in plant height, projected shoot area, and an increase in leaf greening (decrease in senescence index) mediated by drought in our experiments.

## 5 Conclusions

The RGB-based HTP with the Scanalyzer 3D system effectively detected stress in tomatoes caused by a virus (TSWV), a soil-borne fungus (*P. lycopersici*), a root-knot nematode (*M. incognita*), or drought. The senescence index was identified as a reliable indicator of the type of stress affecting plants, namely biotic or abiotic. In fact, the biotic stressors caused early senescence in plants, whereas the drought induced leaf greening. Size-related indices, such as plant height and projected shoot area, effectively described the plant growth stunting caused by stress, whether from drought or from the biotic stresses we examined, without making distinctions among them. This indicates that if the canopy area is the preferred index for a study or application, RGB cameras are undoubtedly the best choice due to their lower cost compared to multispectral or hyperspectral sensors.

It is very likely that more data from RGB, multispectral, or hyperspectral sensors, along with the support of machine learning or artificial intelligence, could lead to even more accurate discrimination of stress types, as observed in olive and almond orchards [55,56]. Complex scenarios involving multiple stresses need further characterization with HTP in tomatoes. While there is evidence that HTP is a valuable tool for crop breeding and management, the future perspective is to apply HTP more extensively in the field, where crops are naturally subjected to various biotic and abiotic stresses that impact both above-ground and below-ground parts of the plants. Furthermore, integrating phenomics and genomics studies will contribute to our understanding of stress responses in tomato, including stress-specific mechanisms of response and adaptation, and may help increase the resilience and sustainability of agriculture [64].

Finally, another critical issue is scaling up HTP procedures from controlled to open-field conditions. To achieve this, multiple challenges must be addressed. For instance, under controlled conditions, plants are often exposed to a single stress, and images are acquired under standardized conditions. In contrast, in the field, crops are subjected to multiple stresses simultaneously, and a variety of environmental conditions may influence the data derived from image analysis. Technology accessibility, immediate data processing, and implementation in the existing farming practices are also issues relevant for scaling up [65].

## Authors contribution

FCe, FCi, MT, LS, and SG conceived the research. MIP and GBu executed the experiment on corky root rot, analyzed the data, prepared the plots and figures, and wrote the manuscript, thus contributing equally to the manuscript. GBa, SG, AR, AC, MDP, and MT carried out the drought experiment. PV and MTM handled the experiment with the root-knot nematode. FCi, LS, and GS conducted the experiment with the tomato spotted wilt virus. AP performed high-throughput phenotyping using the Scanalyzer 3D platform, and SS extracted the numeric data from the RGB images. All the authors revised the manuscript.

## Funding

This research was supported by three projects: i) ‘E-crops: Technologies for Digital and Sustainable Agriculture’ (grant no. ARS01_01136), funded by the Italian Ministry of University and Research (MUR) within the ‘PON Ricerca e Innovazione 2014–2020’ framework, Agrifood area; ii) ‘Agritech National Research Center’ (D.D. 1032 17/06/2022, grant no. CN00000022), funded within the ‘Piano Nazionale di Ripresa e Resilienza’ (PNRR), mission 4 ‘Education and Research’, component 2 ‘From research to business’, investement 1.4 ‘Strengthening research structures and creating national R&D champions on some Key Enabling Technologies’; and iii) ‘ITINERIS: Italian Integrated Environmental Research Infrastructures System (grant no. IR0000032), funded within the PNRR, mission 4, component 2, investment 3.1 ‘Fund for the realization of an integrated system of research and innovation infrastructures’. The latter two projects were funded by the European Union Next Generation EU. This manuscript reflects only the authors’ views and opinions, neither the European Union nor the European Commission can be considered responsible for them.

## Declaration of competing interest

The authors declare that they have no known competing financial interests or personal relationships that could have appeared to influence the work reported in this paper.

## Data Availability

The data collected and used in this study are available in the supplementary material. The code used for analysis is available from the corresponding author, GBu, upon reasonable request.
