# Supplementary material for: High-throughput plant phenotyping identifies and discriminates biotic and abiotic stresses in tomato
Source: Plant Phenomics. 2025 Sep 30;7(4):100124. doi: 10.1016/j.plaphe.2025.100124 (PMC13109330; doi:10.1016/j.plaphe.2025.100124)
Supplement: Multimedia component 1 [file mmc1.pdf]

SUPPLEMENTAL MATERIAL OF THE ARTICLE:

High-throughput plant phenotyping identifies and discriminates biotic and abiotic stresses in tomato

Maria Isabella Prigigallo, Giovanni Bubici, Giorgia Batelli, Antonello Costa, Monica De Palma, Maria Teresa Melillo, Angelo Petrozza, Alessandra Ruggiero, Giorgia Sportelli, Stephan Summerer, Pasqua Veronico, Francesco Cellini, Marina Tucci, Livia Stavolone, Stefania Grillo, Fabrizio Cillo

Table S1. Tomato genotypes used in the five experiments of this research.

Genotype	TSWV	CRR	RKN	Drought (spring)	Drought (fall)
UC82	x[a]	x[a]	x	x	x
Faber F_1	x[a]			x	
Impact F_1	x[a]				
Dobler F_1	x[b]			x	
Moboglan (LA2824)		x[b]			
San Marzano Nano			x	x	x
Regina di Fasano			x	x	
Red Setter				x	x
M82					x
Cerise				x	
Torremaggiore				x	
770P				x	
990P				x	
Seccagno				x	
Mariner F_1				x	
Eventus F_1				x	
SV48840TM				x	
Taylor F_1				x	

[a] Susceptible control.
[b] Resistant control.

Table S2. Characteristics of the genotypes used in this study.

Genotype	Plant growth habitus	Fruit shape	Provider
770P	Determinate	Round, apex	IBBR Portici seed collection
990P	Semi-determinate	Round	IBBR Portici seed collection
Cerise	Determinate	Obovoid	Semiorto Sementi
Dobler F_1	Determinate	Blocky	ISI Sementi
Eventus F_1	Determinate	Elongated	Seminis-Bayer
Faber F_1	Determinate	Blocky	ISI Sementi
Impact F_1	Determinate	Blocky	ISI Sementi
M82	Determinate	Rectangular	IBBR Portici seed collection
Mariner F_1	Determinate	Elongated	ISI Sementi
Moboglan (LA2824)	Indeterminate	Round	TGRC
Red Setter	Determinate	Rectangular	IBBR Portici seed collection
Regina di Fasano	Determinate	Obovoid	Sementi Larosa
San Marzano nano	Determinate	Elongated	Semiorto Sementi
Seccagno	Determinate	Rectangular	Regione Campania
SV48840TM	Determinate	Blocky	Seminis-Bayer
Taylor F_1	Determinate	Elongated	BASF
Torremaggiore	Determinate	Round	Semiorto Sementi
UC82	Determinate	Obovoid	Semiorto Sementi

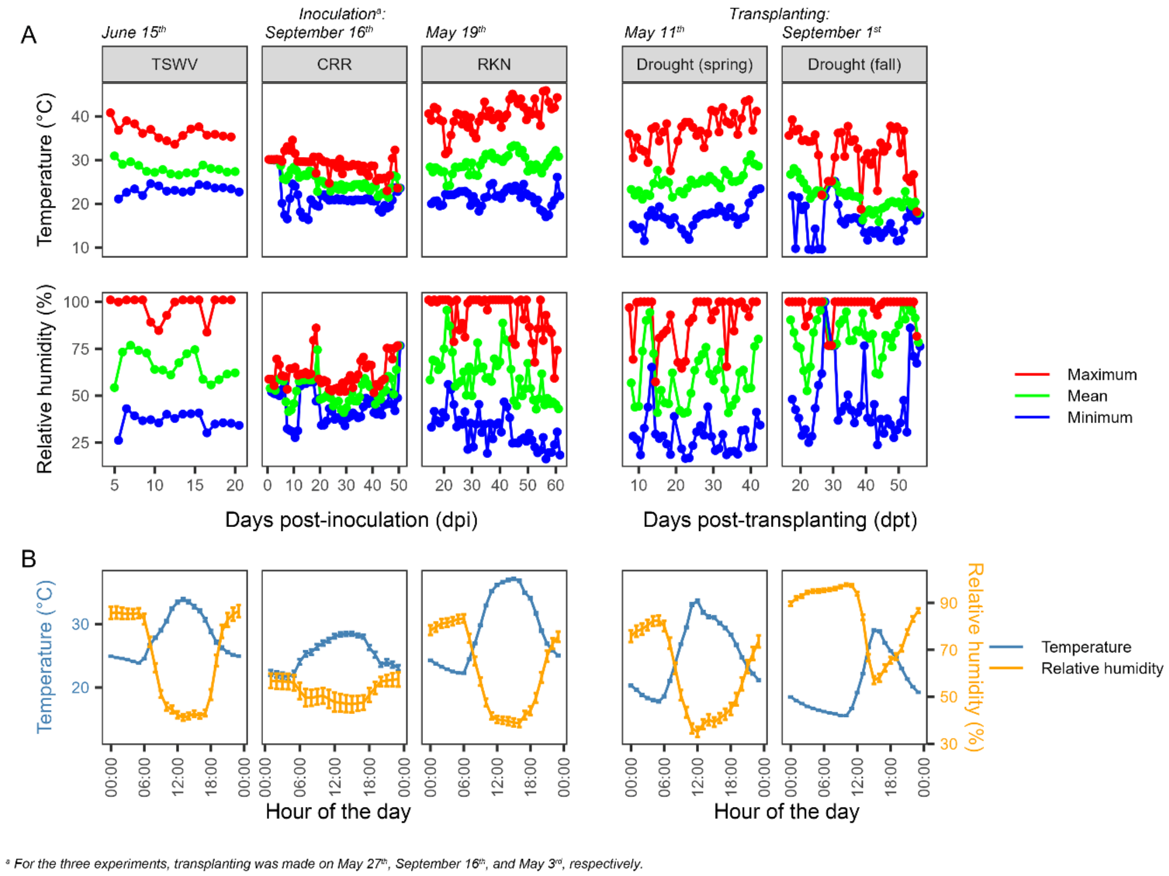

Figure S1. Environmental parameters recorded throughout five experiments on tomato plants in the glasshouse (A) and mean values by the hour of the day (B).

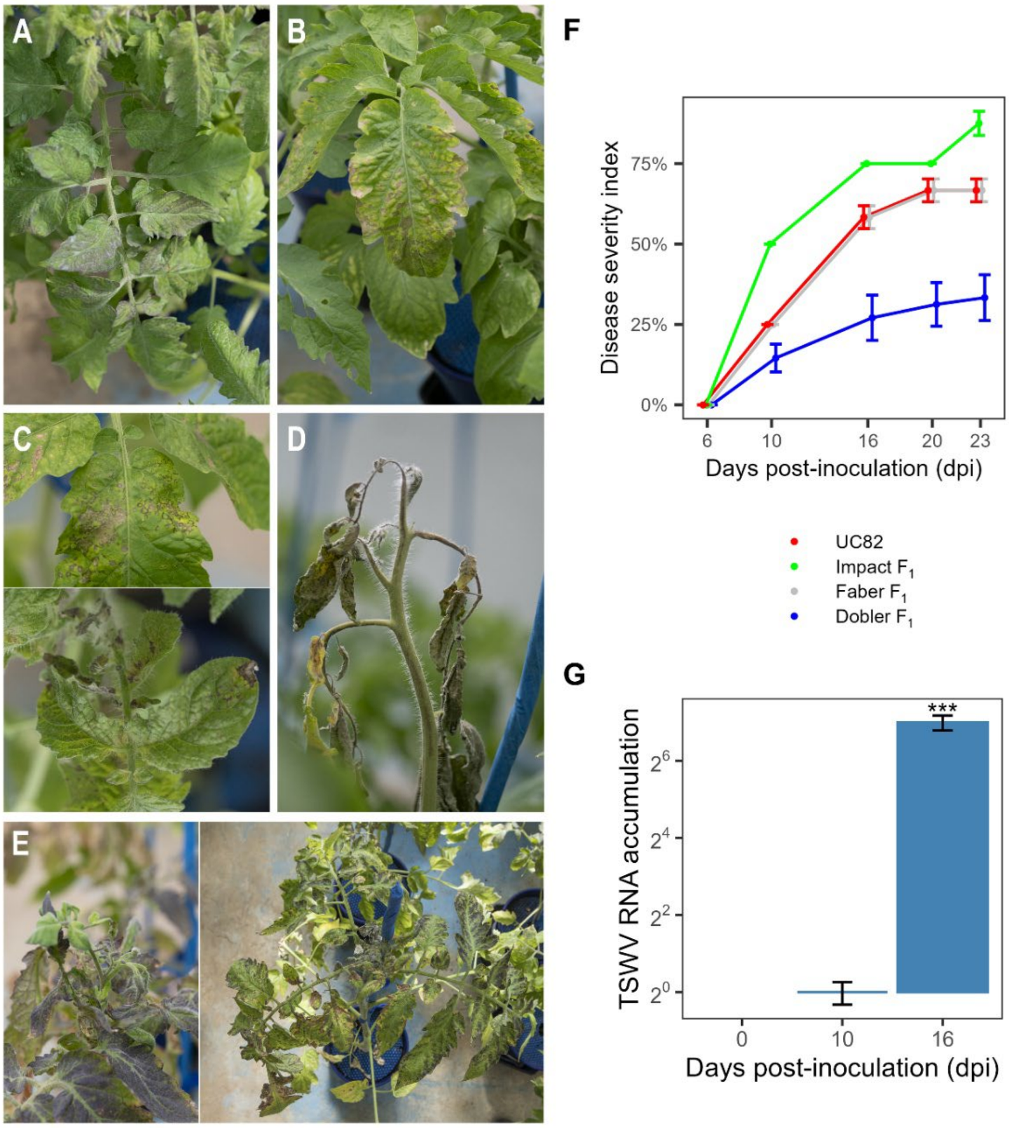

Figure S2. Development of viral disease symptoms and tomato spotted wilt virus (TSWV) disease. Symptoms of TSWV T-1012 on different tomato genotypes at 16 days post-inoculation (dpi): UC82 (A); Faber F_1 (B); Impact F_1 (C); Dobler F_1 (D). Symptoms of TSWV T-1012 on Impact F_1 (left in E) and UC82 at 36 dpi (right in E). Progress over time of the severity of symptoms caused by TSWV on four artificially inoculated tomato genotypes (F). Error bars indicate the standard error of the mean (n=12). Reverse transcriptase quantitative PCR (RT-qPCR) quantification of TSWV RNA accumulation in tomato plants cv. UC82 at 10 and 16 dpi (G). The average value of TSWV RNA at 10 dpi is set as unity (1), and viral RNA accumulation at 16 dpi is then calculated relatively. Error bars indicate the standard error of the mean (n=3). Asterisks indicate a significant difference compared to 0 dpi according to the Student's *t*-test (***=$P<0.001$).

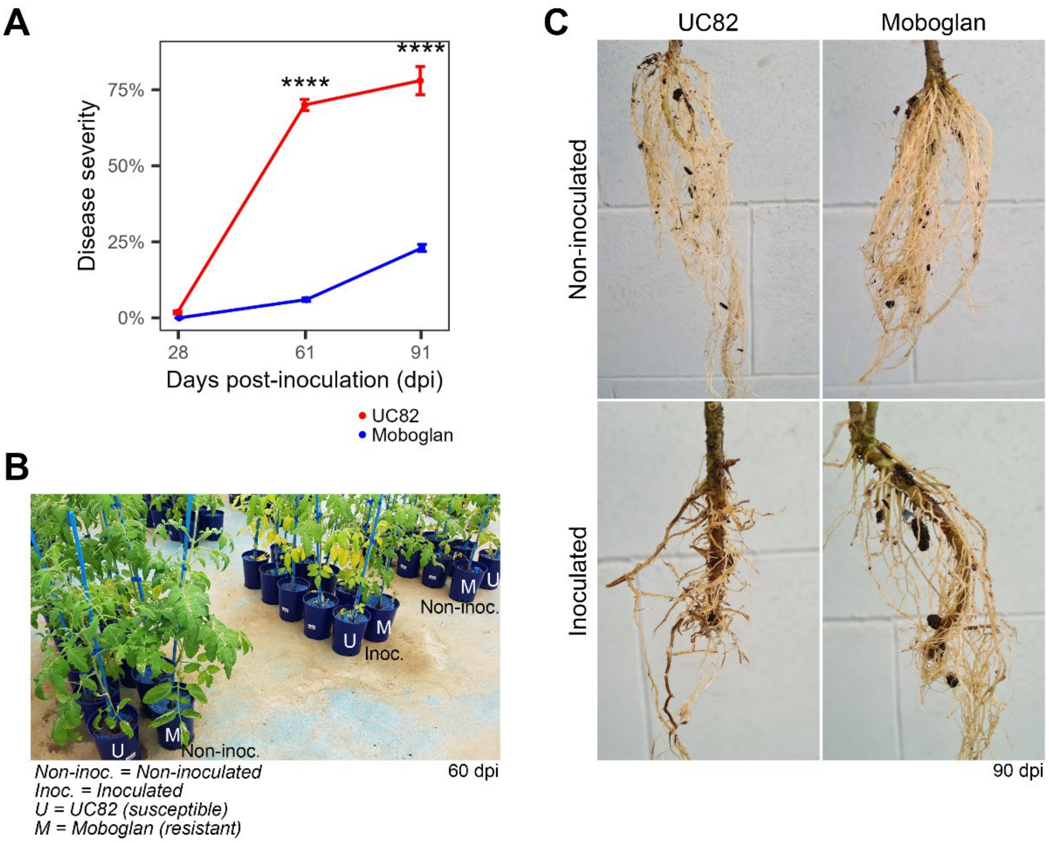

Figure S3. Progress over time of corky root severity on two tomato genotypes artificially inoculated with *Pseudopyrenochaeta lycopersici* (A). Error bars indicate the standard error of the mean (n=15). Asterisks indicate a significant difference between control and stressed plants according to the Student's *t*-test (****=P<0.0001). Tomato plants 'UC82' and 'Moboglan' inoculated or not with the pathogen: leaf yellowing is evident on inoculated plants (B). Corky root symptoms on roots of the susceptible ('UC82') and resistant ('Moboglan') tomato genotypes (C).

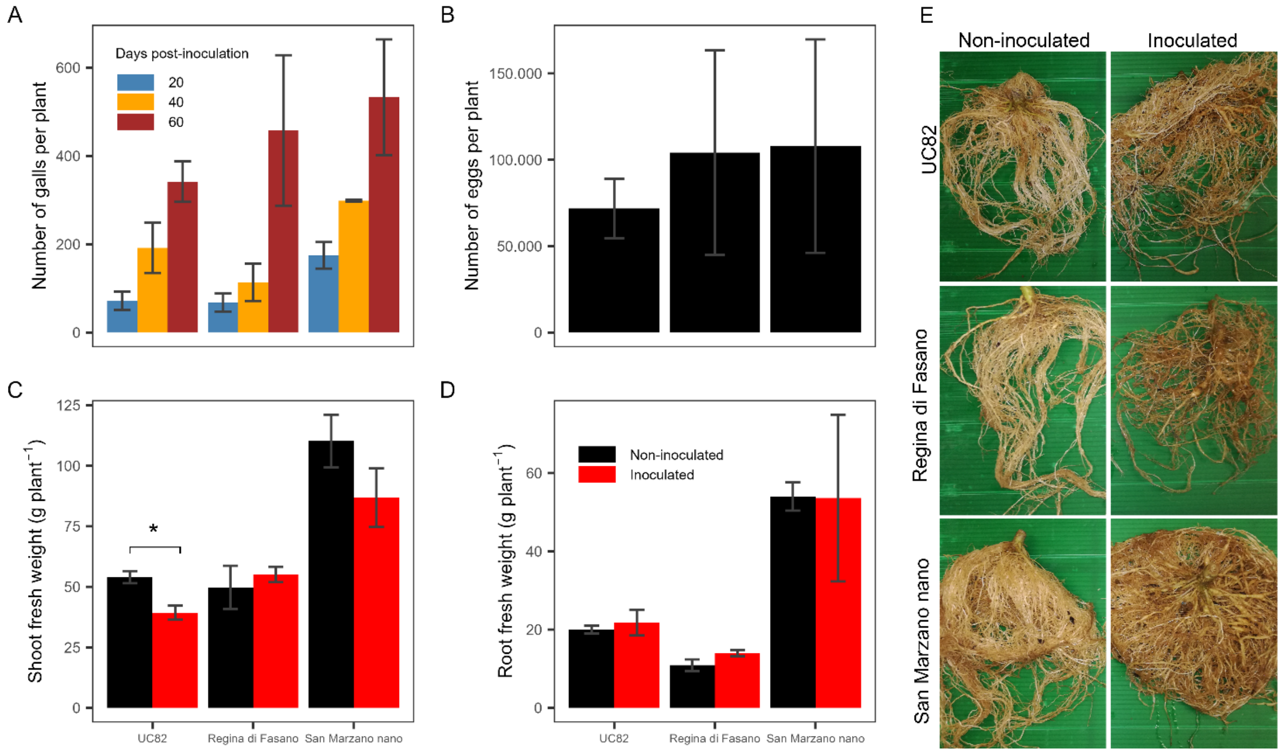

Figure S4. Effect of the root-knot nematode *Meloidogyne incognita* infection on three tomato genotypes, ‘UC82’, ‘Regina di Fasano’, and ‘San Marzano nano’. Progress over time of the nematode infection (A). In B-D, data from 60 days post-inoculation (dpi) are reported. In the plots (A-D), error bars indicate the standard error of the mean (n=3 in A and n=6 in B-D). Asterisk indicates a significant difference according to Student’s *t*-test ($P<0.05$). Root systems of the three tomato genotypes at 60 dpi: galls are evident on the inoculated roots (E).

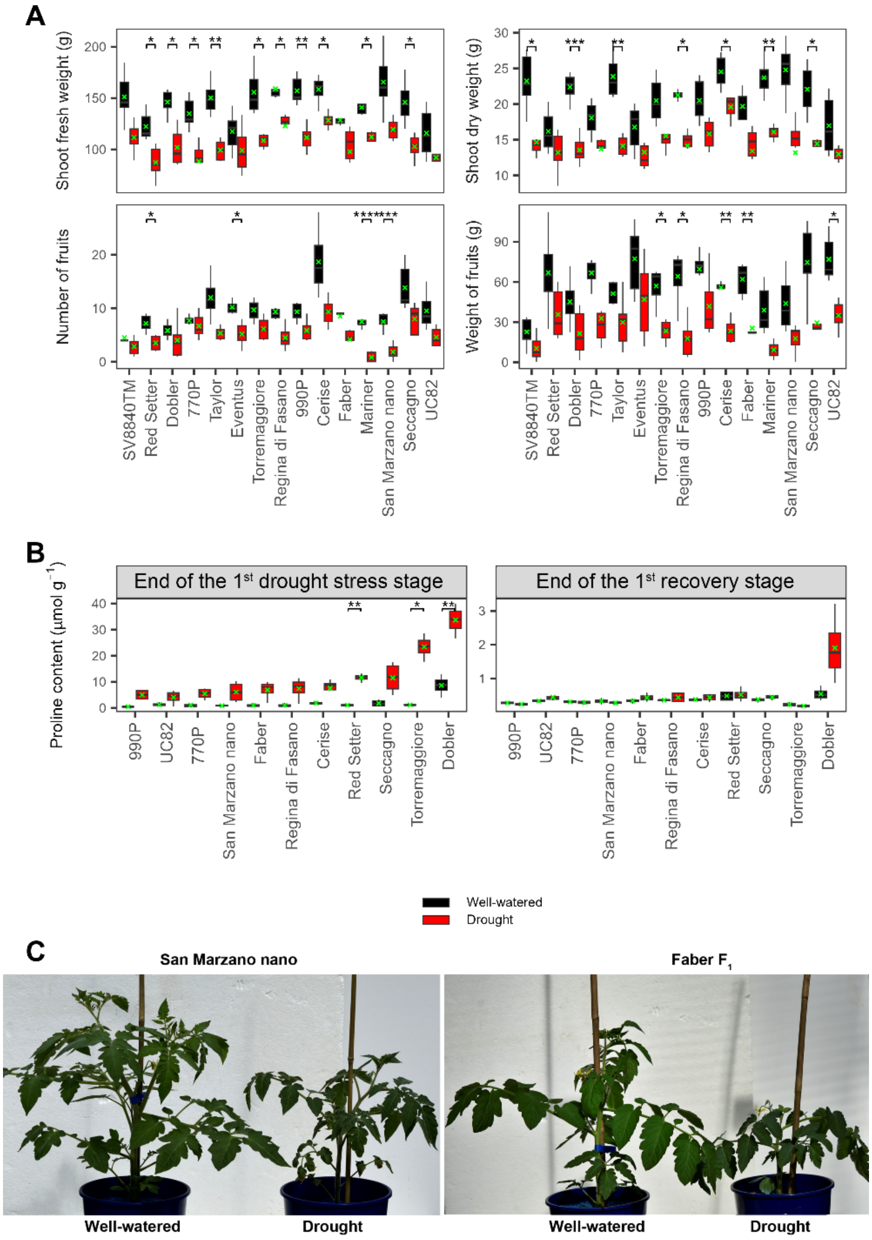

Figure S5. Biometric parameters (shoot fresh and dry weights, fruit number, and weight) determined at the end of the drought experiment conducted in the spring of 2021 on 15 tomato genotypes subjected or not to two cycles of drought stress and recovery (A). Leaf proline content at the end of the first drought stress event and the following recovery stage (B). Error bars indicate the standard error of the mean (n=6). Asterisks indicate a significant difference between control and stressed plants according to the Student's *t*-test (*=*P*<0.05; **=*P*<0.01; ***=*P*<0.001; ****=*P*<0.0001). Growth stunting of two tomato varieties after two cycles of drought stress and recovery (C).

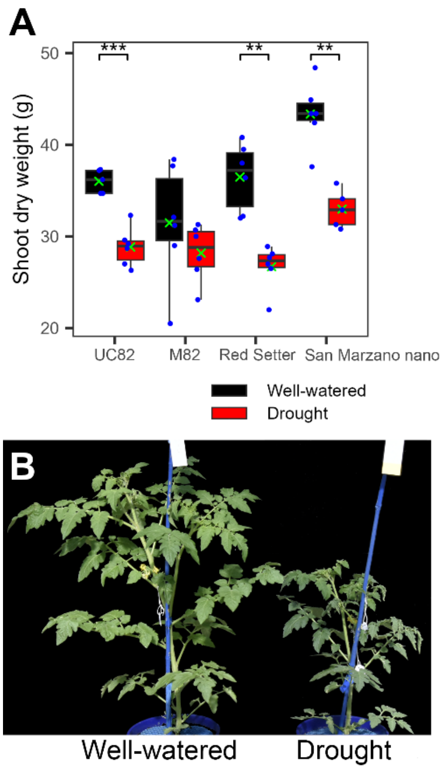

Figure S6. Shoot dry weight determined at the end of the drought experiment conducted in the fall of 2021 on four tomato genotypes subjected or not to two cycles of drought stress and recovery (A). Error bars indicate the standard error of the mean (n=15). Asterisks indicate a significant difference between control and stressed plants according to the Student's *t*-test (**=*P*<0.01; ***=*P*<0.001). Growth stunting of tomato cv. UC82 after two cycles of drought stress and recovery (B).

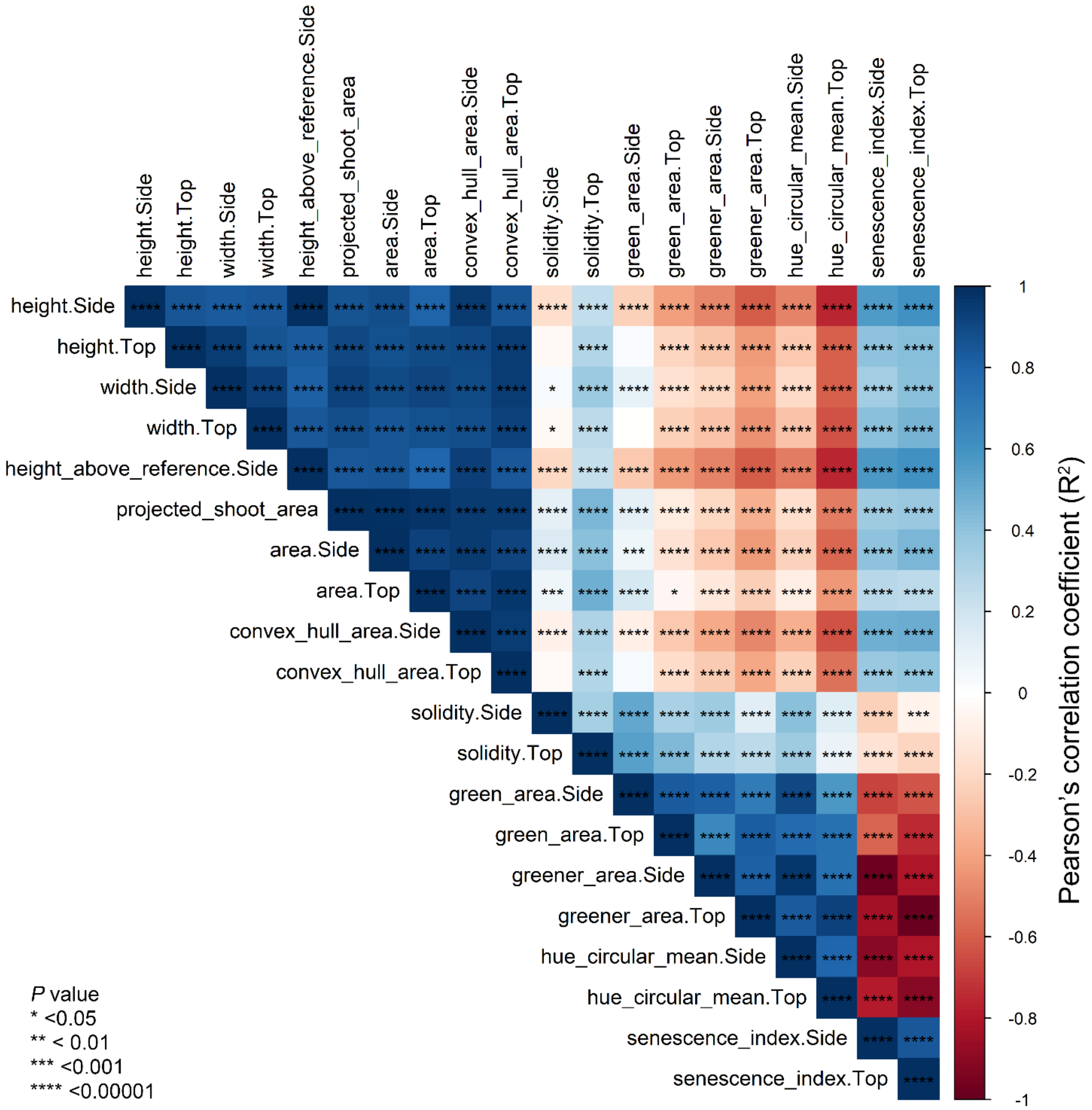

Figure S7. Pearson's correlation matrix of 20 High-Throughput Phenotyping (HTP) indices computed with the whole dataset (2883 observations and 20 variables) of five stresses: tomato spotted wilt virus (TSWV), corky root rot (CRR), root-knot nematode (RKN), and drought conducted in spring and fall.

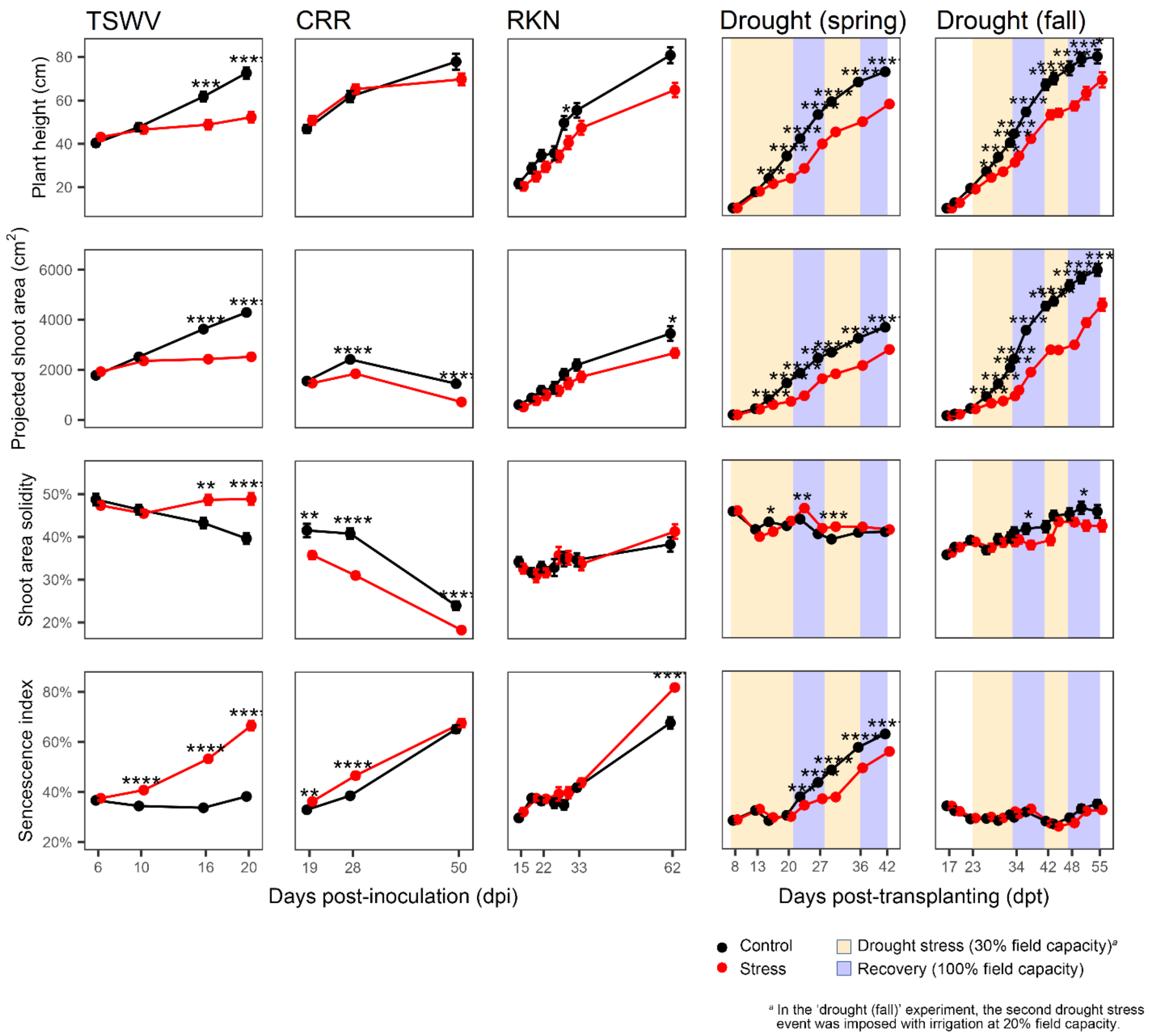

Figure S8. Progress over time of four indices on tomato plants (average of all genotypes assayed) challenged separately with five stresses: tomato spotted wilt virus (TSWV), corky root rot (CRR), root-knot nematode (RKN), and drought conducted in spring and fall. Error bars indicate the standard error of the mean. Asterisks indicate a significant difference between control and stressed plants according to the Student's *t*-test (*=P<0.05; **=P<0.01; ***=P<0.001; ****=P<0.0001).

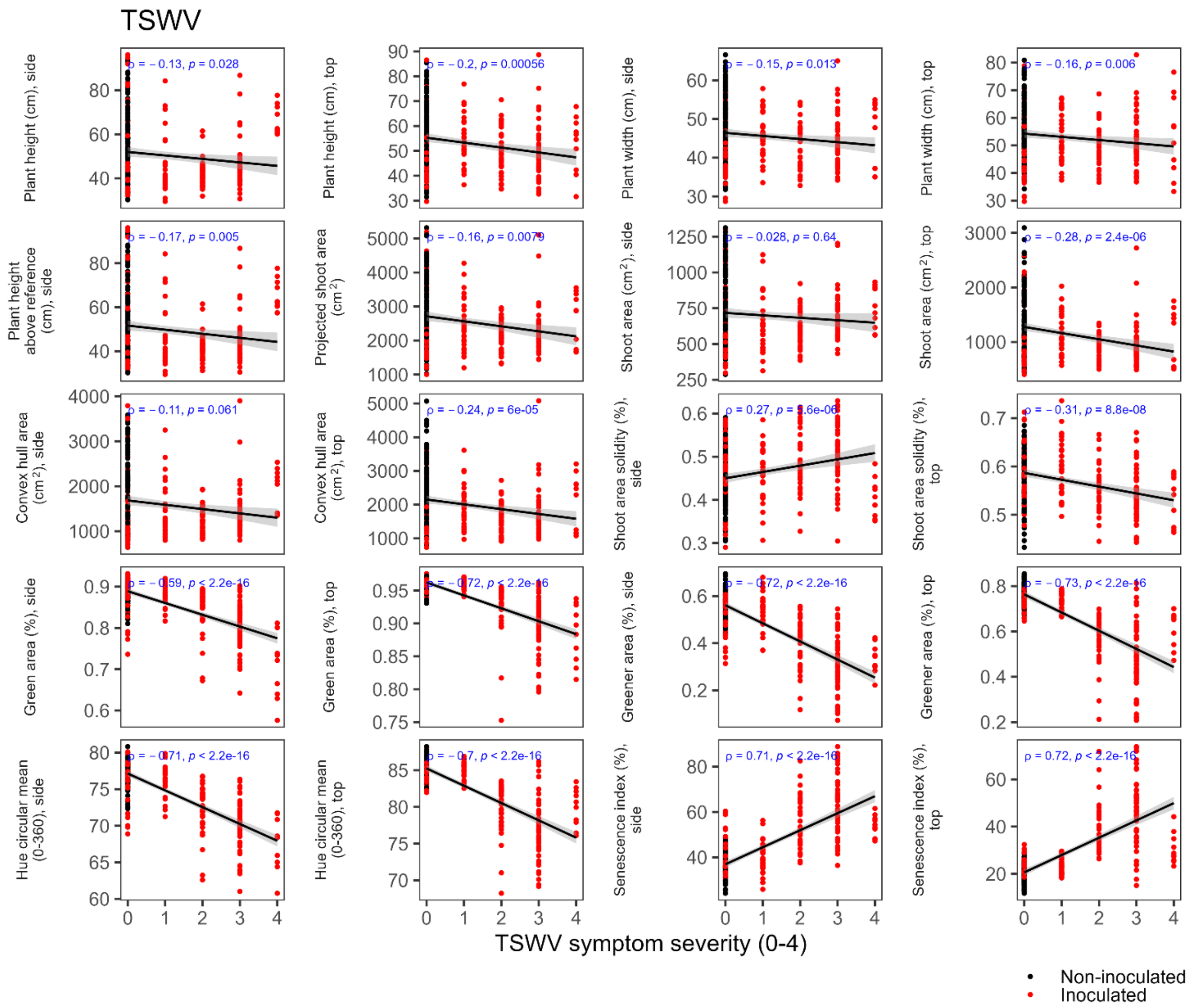

Figure S9. Spearman's correlations between 20 High-Throughput Phenotyping (HTP) indices and symptom severity of tomato spotted wilt virus (TSWV). The shadow of the regression line shows the confidence interval.

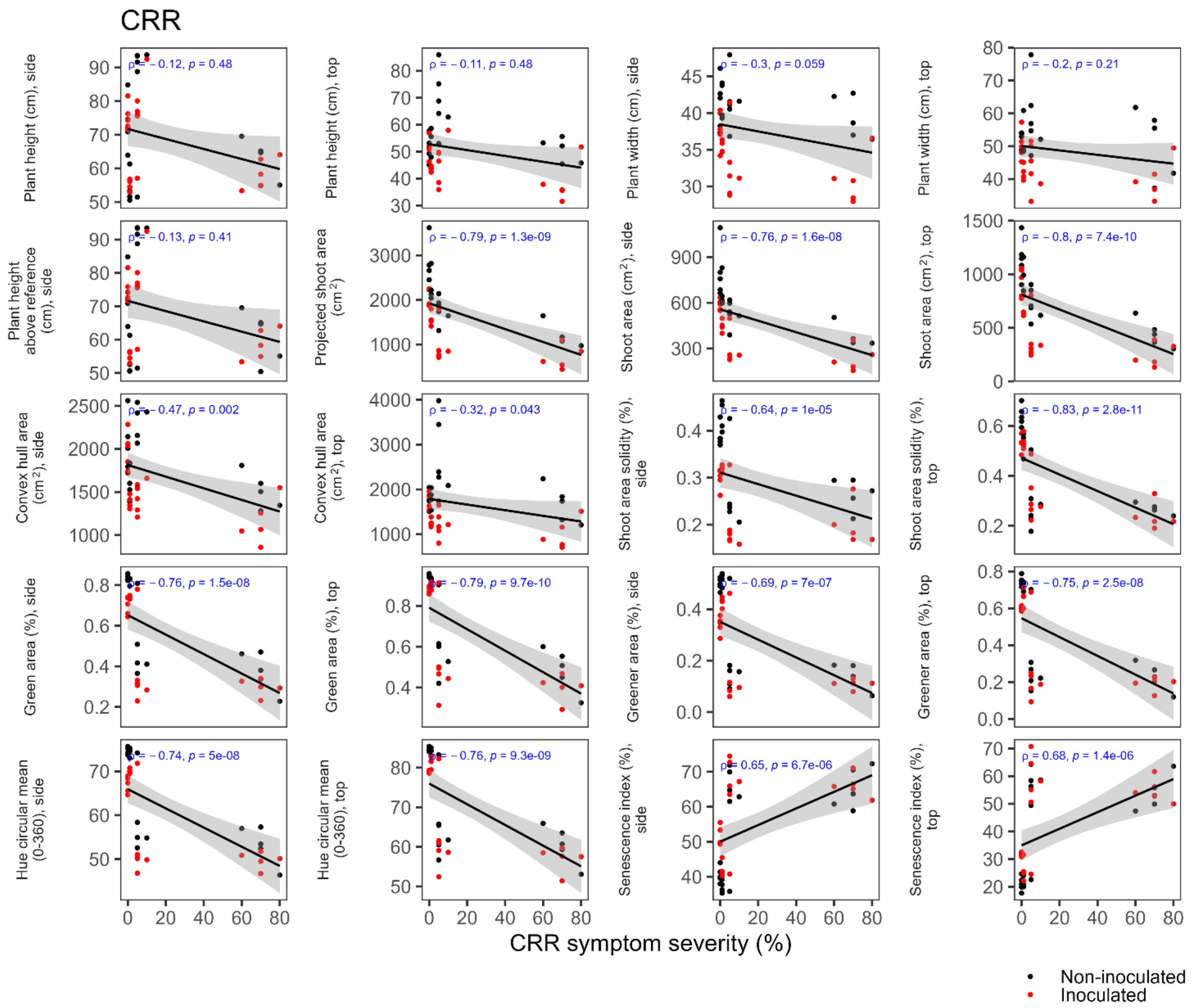

Figure S10. Spearman's correlations of 20 High-Throughput Phenotyping (HTP) indices with corky root rot (CRR) symptom severity. The shadow of the regression line shows the confidence interval.

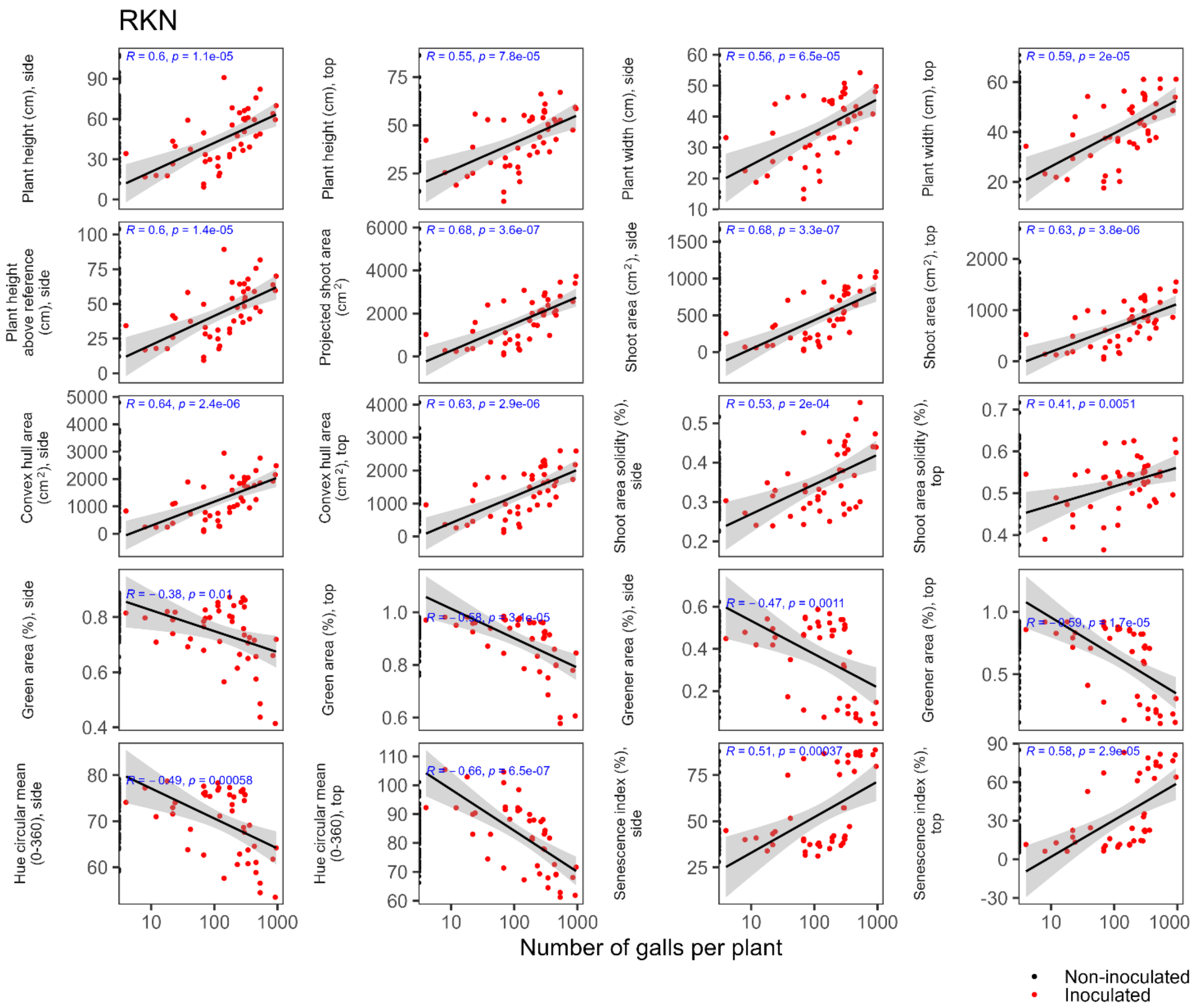

Figure S11. Pearson's correlations between 20 High-Throughput Phenotyping (HTP) indices and the number of root galls caused by the root-knot nematode (RKN). The shadow of the regression line shows the confidence interval.

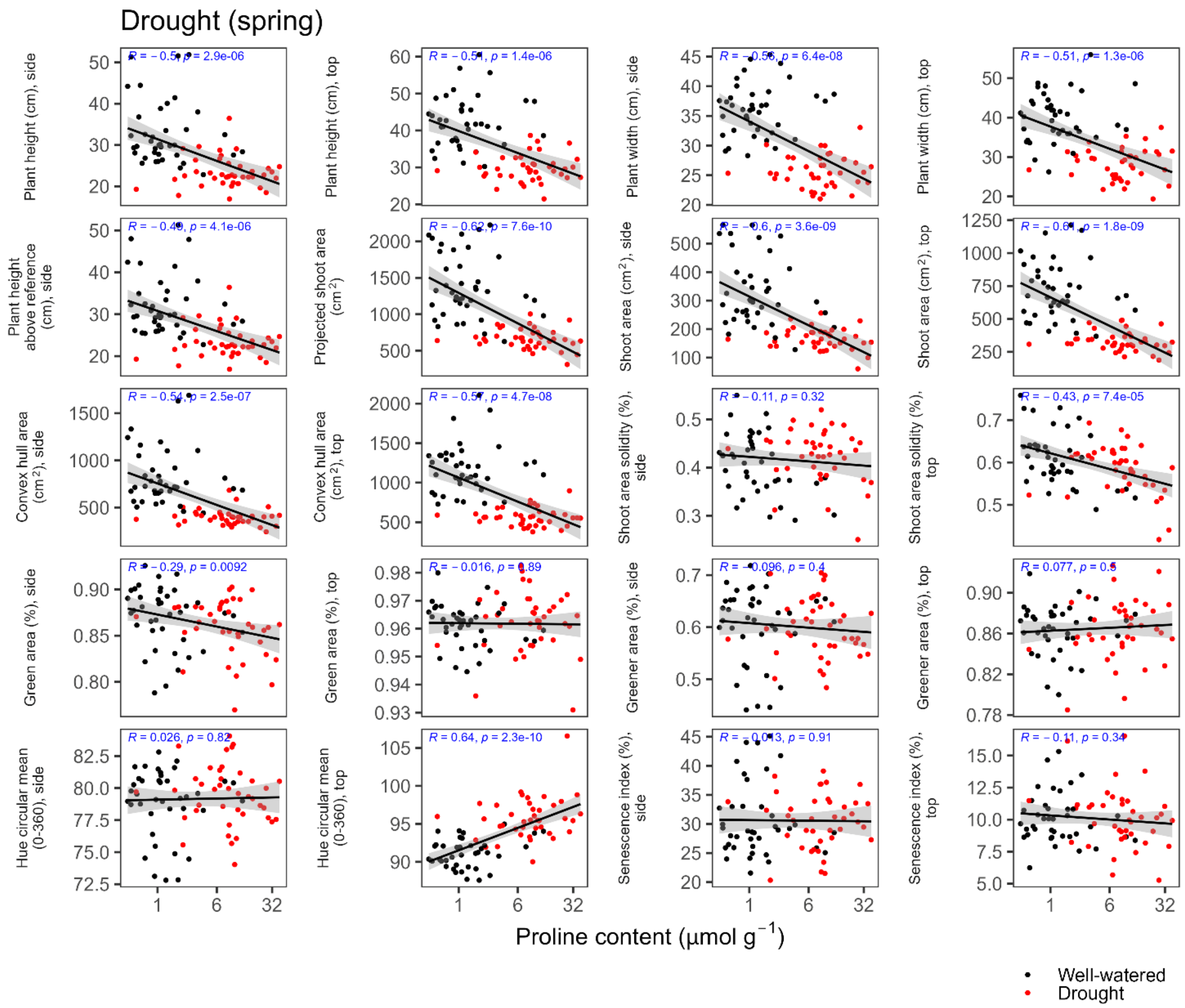

Figure S12. Pearson's correlations of 20 High-Throughput Phenotyping (HTP) indices with proline content of plants at 20 days post-transplanting (the end of the first drought event) in a drought experiment conducted in the spring. The shadow of the regression line shows the confidence interval.

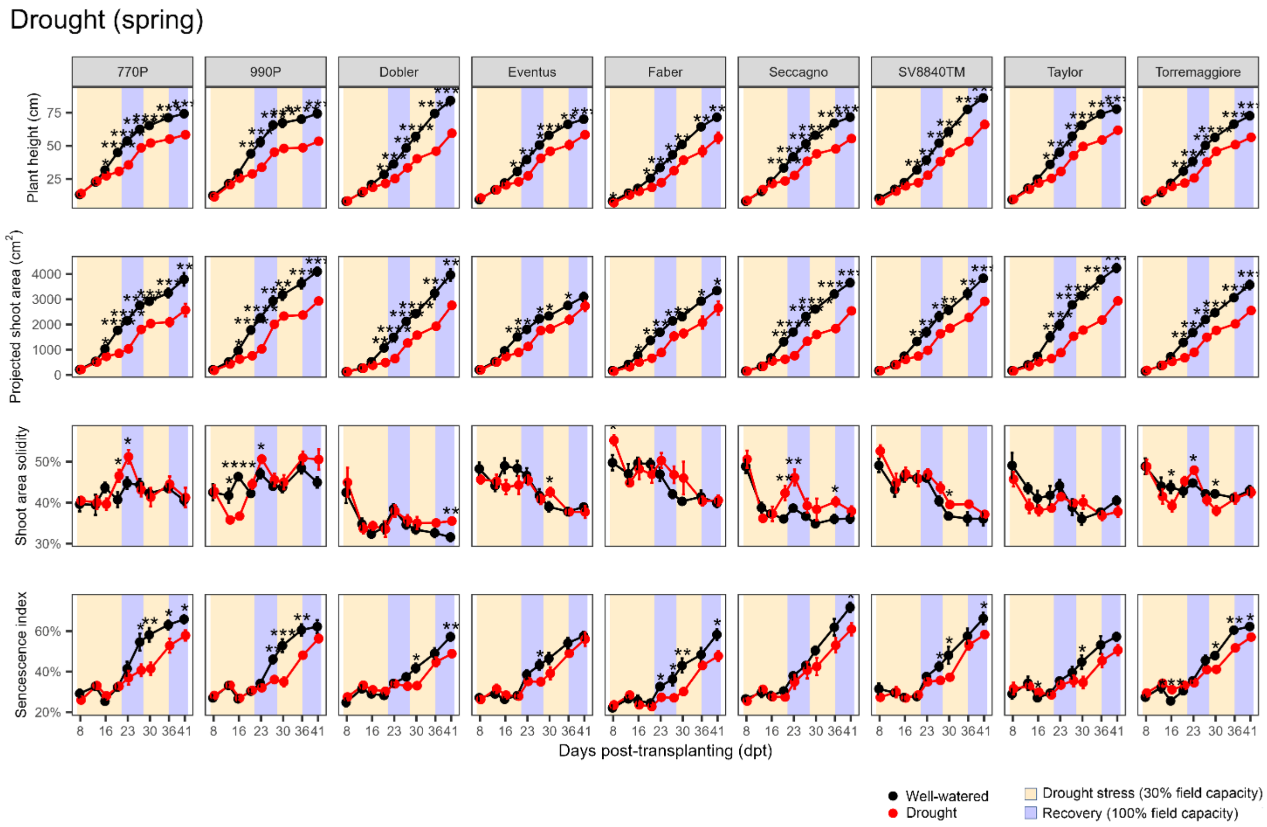

Figure S13. Progress over time of four high-throughput phenotyping indices on nine tomato genotypes subjected to two cycles of drought stress and recovery in the spring of 2021. Error bars indicate the standard error of the mean (n=6). Asterisks indicate a significant difference between control and stressed plants according to the Student's *t*-test (*=*P*<0.05; **=*P*<0.01; ***=*P*<0.001; ****=*P*<0.0001). Other genotypes are shown in Figure 6.

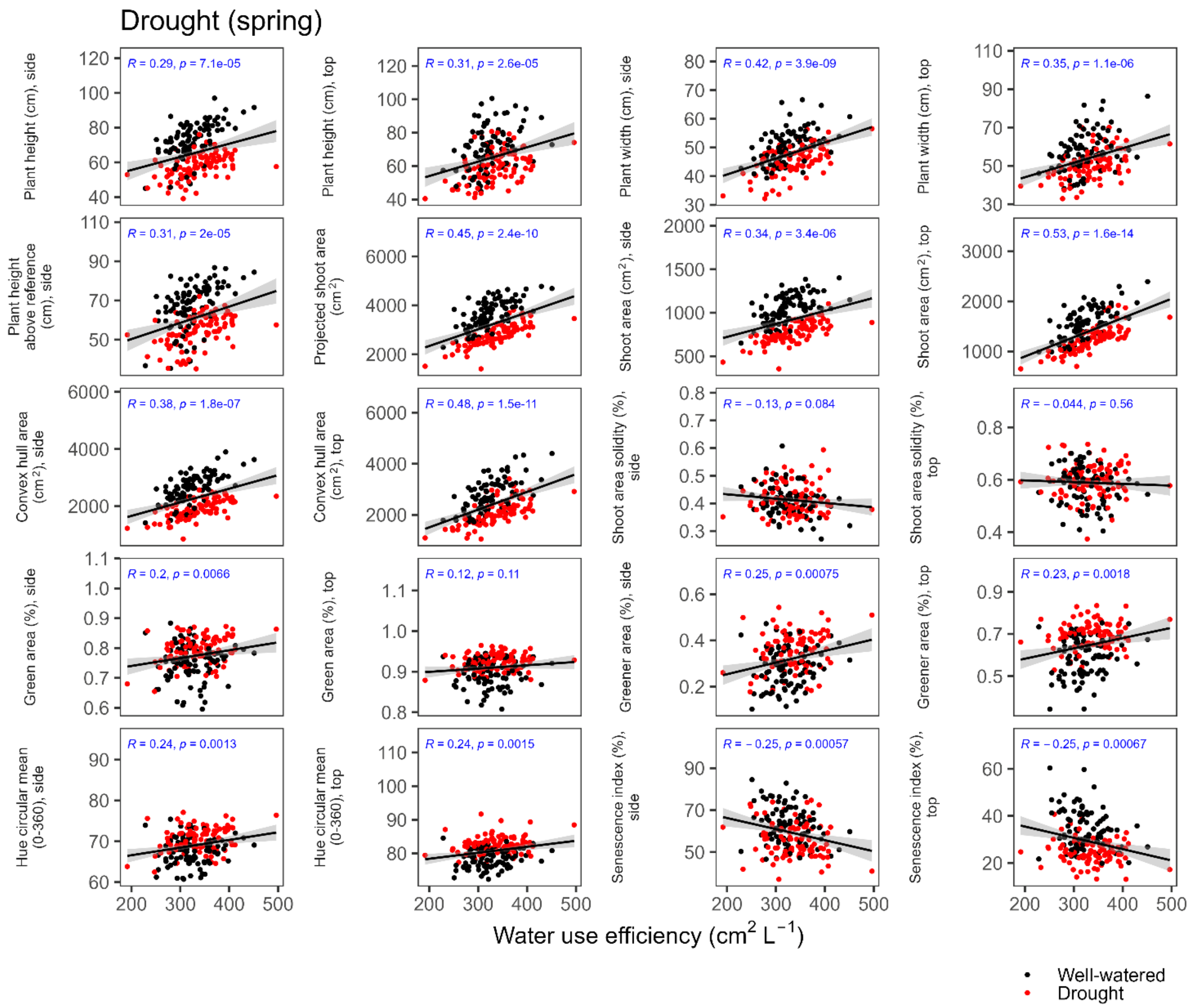

Figure S14. Pearson's correlations of 20 High-Throughput Phenotyping (HTP) indices with the water use efficiency at the end of the experiment in the drought experiment conducted in the spring. The shadow of the regression line shows the confidence interval.

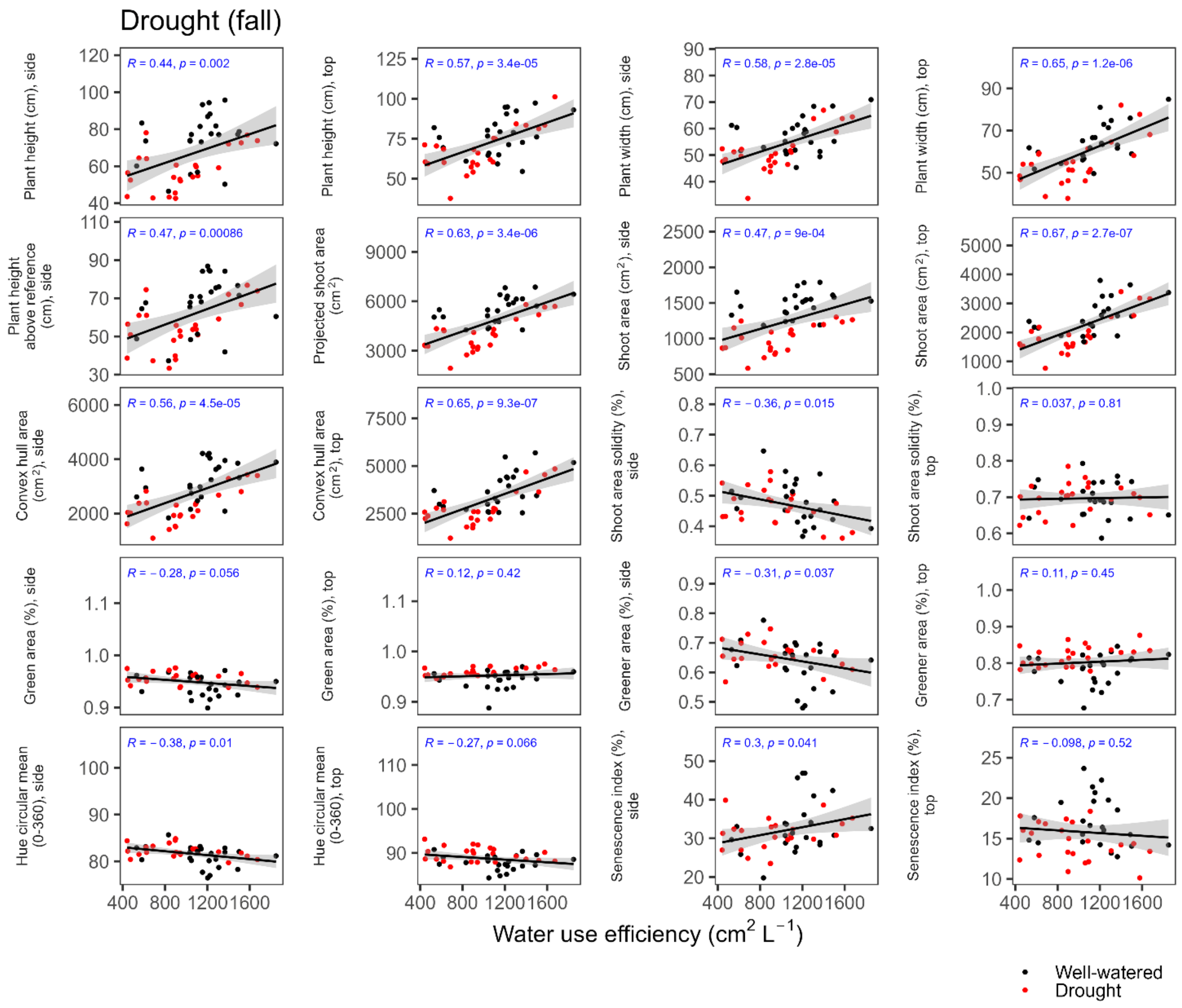

Figure S15. Pearson's correlations of 20 High-Throughput Phenotyping (HTP) indices with the water use efficiency at the end of the experiment in the drought experiment conducted in the fall. The shadow of the regression line shows the confidence interval.
